# Notch signalling is a potential resistance mechanism of progenitor cells within patient‐derived prostate cultures following ROS‐inducing treatments

**DOI:** 10.1002/1873-3468.13589

**Published:** 2019-09-17

**Authors:** John R. Packer, Adam M. Hirst, Alastair P. Droop, Rachel Adamson, Matthew S. Simms, Vincent M. Mann, Fiona M. Frame, Deborah O'Connell, Norman J. Maitland

**Affiliations:** ^1^ Cancer Research Unit Department of Biology University of York UK; ^2^ Department of Physics York Plasma Institute University of York UK; ^3^ Faculty of Biological Sciences University of Leeds UK; ^4^ Department of Urology Castle Hill Hospital (Hull and East Yorkshire Hospitals NHS Trust) Cottingham UK

**Keywords:** Low temperature plasma, Notch signalling, progenitor cells, prostate cancer, reactive oxygen species, Therapy resistance

## Abstract

Low Temperature Plasma (LTP) generates reactive oxygen and nitrogen species, causing cell death, similarly to radiation. Radiation resistance results in tumour recurrence, however mechanisms of LTP resistance are unknown. LTP was applied to patient‐derived prostate epithelial cells and gene expression assessed. A typical global oxidative response (AP‐1 and Nrf2 signalling) was induced, whereas Notch signalling was activated exclusively in progenitor cells. Notch inhibition induced expression of prostatic acid phosphatase (PAP), a marker of prostate epithelial cell differentiation, whilst reducing colony forming ability and preventing tumour formation. Therefore, if LTP is to be progressed as a novel treatment for prostate cancer, combination treatments should be considered in the context of cellular heterogeneity and existence of cell type‐specific resistance mechanisms.

## Graphical abstract

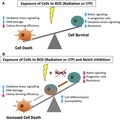

## Abbreviations

**ADT**, androgen‐deprivation therapy

**CB**, committed basal

**LTP**, low temperature plasma

**PAP**, prostatic acid phosphatase

**qRT‐PCR**, quantitative reverse transcription PCR

**RNS**, reactive nitrogen species

**ROS**, reactive oxygen species

**SC**, stem cell

**TA**, transit amplifying

There is much interest in the potential for Low Temperature Plasma (LTP) to be a novel focal therapy for cancer 1. Therefore, this study set out to determine the mechanism of action of LTP in a near patient model of prostate cancer. LTP is formed by application of high voltage across a gas‐flow, causing removal and subsequent acceleration of gas molecule electrons into surrounding atoms and molecules. A reactive cascade of interconverting neutral and charged species is created in the plasma effluent, alongside the emission of UV radiation. Mixing of LTP with gas molecules in the air produces high concentrations of reactive oxygen and nitrogen species (ROS and RNS) 2, 3 that can cause oxidative stress, DNA damage 1, 2 protein oxidation 4 and lipid peroxidation in cells 5. LTP‐based treatment of cancer cells is being considered for a variety of malignancies 6, including prostate cancer 1. However, before this mode of treatment can be developed for clinical use, the full mechanism of action, and potential mechanisms of resistance, needs to be elucidated.

Oxidative stress, as induced by LTP, is defined by an imbalance of ROS over cellular antioxidants. Current prostate cancer treatments such as radiotherapy and photodynamic therapy generate ROS, which act as their primary therapeutic agent 7. Oxidative response occurs rapidly through the buffering effects of enzymatic activities and transcriptional programs. Gene expression following exposure to reactive species generated by ionising radiation, is mediated by multiple transcription factors including Nrf2 and AP‐1 5.

Previous studies, which applied LTP treatment to established cancer cell lines, have shown that LTP induces apoptotic cell death 2, 3, 4. However, in primary prostate basal epithelial cultures LTP can also induce autophagy and necrosis 1. Critically, a resistant population of viable cells remains after exposure to LTP; understanding how these cells survive should allow optimisation of LTP‐induced cell death.

Here, we present an analysis of the signalling events immediately following LTP treatment of primary prostate basal epithelial cells, directly cultured from normal, benign and cancer tissues from individual patients. Whilst we observed the predicted activation of AP‐1 and Nrf2 oxidative stress response signalling pathways, we describe for the first time, activation of Notch signalling by LTP. Significantly, this occurred in the progenitor cell population only, and not the more differentiated cells within the cultures. Notch is considered to be a regeneration signal and marker of self‐renewing stem cell (SC) populations. Activation of this pathway has implications for potential resistance to LTP whilst the selective use of a clinically available Notch inhibitor has the potential to increase the potency of ROS/RNS species for prostate cancer treatment. Indeed, Notch inhibition using gamma‐secretase inhibitors induced expression of prostatic acid phosphatase (PAP), indicative of cell differentiation, and a combination of Notch inhibition with radiation resulted in reduced colony forming ability compared to Notch inhibition or radiation alone. In addition, Notch inhibition reduced or prevented tumour formation *in vivo*. Before LTP can progress to the clinic, the mechanisms of resistance need to be elucidated alongside proposed combination treatment strategies to overcome these. Here, we propose Notch signalling as a major resistance mechanism that could be exploited for therapeutic purposes.

## Materials and methods

### Culture of primary prostate basal epithelial cells

**Figure 1:**
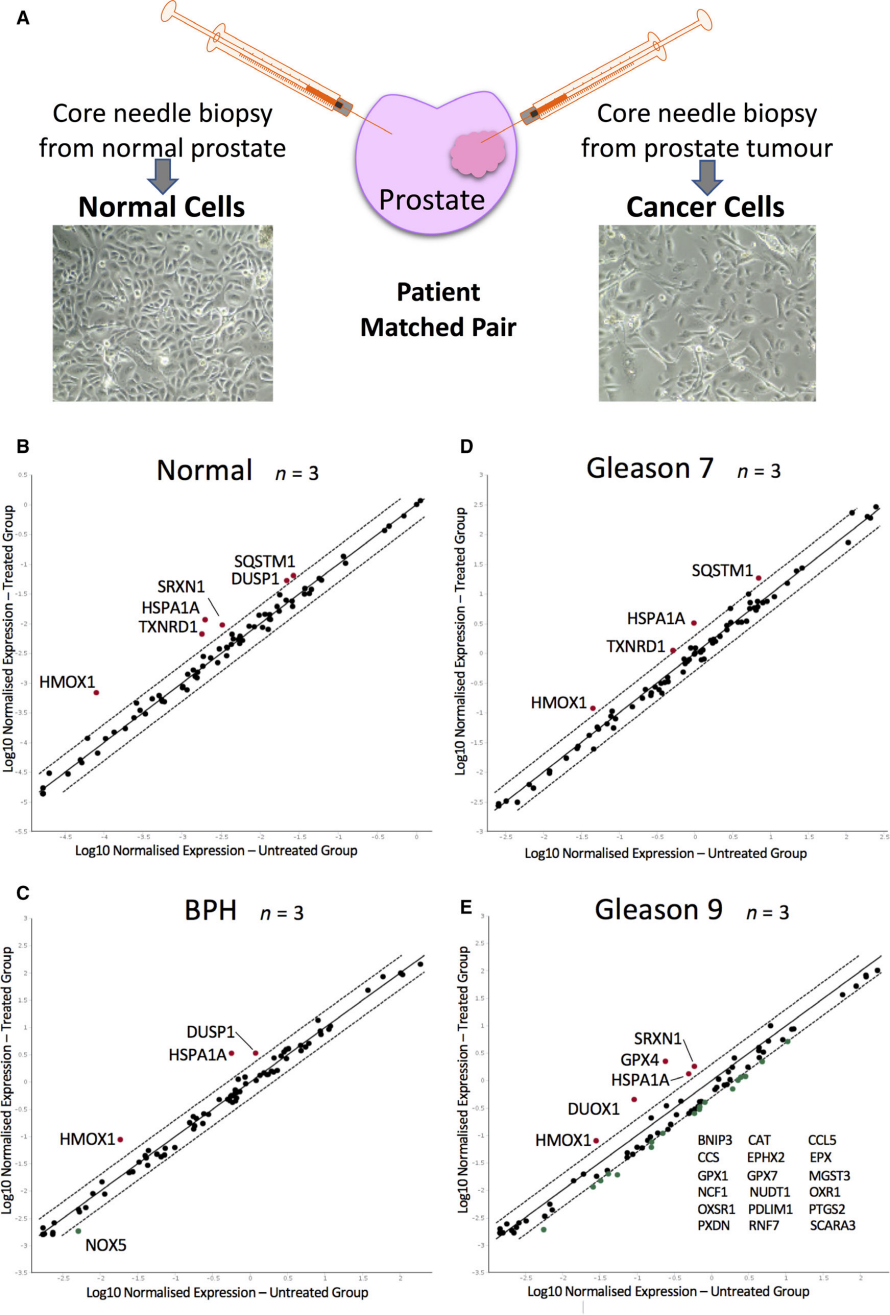
LTP activates oxidative stress responsive gene expression in primary prostate epithelial cell cultures. (A) Retrieval of targeted needle biopsies from prostate tumour and from adjacent non‐cancerous (normal) tissue. Average gene expression of 12 patient cultures of confirmed tissue pathology; (B) Normal (*n* = 3), (C) BPH (*n* = 3), (D) Gleason 7 (*n* = 3), (E) Gleason 9 (*n* = 3) at 2 h post 3 min LTP treatment. Each gene is represented by a single dot; black – unchanged, red – upregulated ≥ 2‐fold and green – downregulated ≥ 2‐fold. The solid black diagonal line represents no change between untreated to treated expression, the flanking dashed lines – a ≥ 2‐fold expression change.

For matched organ‐confined Gleason 7 prostate cancer and normal cells, tissue was obtained by needle biopsy immediately following radical prostatectomy (Fig. 1A). Biopsy sites were informed by previous pathology, MRI imaging and palpation. Benign Prostatic Hyperplasia (BPH) and Gleason 9 tissue were obtained through trans‐urethral resection of the prostate. Tissues were transported in RPMI‐1640 (Gibco, Waltham, MA, USA) supplemented with 5% FCS (Gibco) and 100 U·mL^−1^ antibiotic/anti‐mycotic solution (Gibco) at 4 °C and were processed, as previously described 8, within 6 h of surgery. All tissue was obtained with full ethical permission and informed consent (REC ref 07/H1304/121). Patient identities were anonymised at source. Primary cells were grown on BioCoat™ Collagen I coated 10 cm dishes (Corning Inc, Corning, NY, USA) in SC media, based upon keratinocyte serum free media (Gibco) supplemented with l‐glutamine (Gibco), SC factor, granulocyte macrophage colony stimulating factor, cholera toxin, bovine pituitary extract, epidermal grown factor (Gibco) and leukaemia inhibitory factor 9. Cells were fed every other day and sub‐cultured when confluent using 1× Trypsin‐EDTA (Gibco). No cultures used in this study were passaged more than five times, to limit divergence from the original donated tissue. Primary cells were cultured in the presence of irradiated (murine) STO feeder cells and no antibiotic/anti‐mycotics were used to maintain the cultures. STOs were depleted before any LTP treatments to remove any mouse cell artefacts.

### Selection of basal epithelial sub‐populations

Primary prostate epithelial cell cultures are heterogeneous and subpopulations can be separated using rapid collagen adherence due to differential expression of α_2_β_1_integrin 10. Basal epithelial cells were plated onto BioCoat™ Collagen I coated 10 cm dishes (Corning) that had been blocked for 1 h at 37 °C in 0.3% BSA (Sigma, Gillingham, UK) PBS (Gibco)(heat treated at 80 °C for 10 min and filtered). After 5 min of incubation at 37 °C, the rapidly adherent α_2_β_1_integrin^hi^ cells [SC and Transit Amplifying (TA) populations] were washed in PBS and harvested by trypsinisation. The non‐adherent α_2_β_1_integrin^lo^ cells [Committed Basal (CB) population] were collected from the plates after the 5‐min incubation and in the PBS washes. Cells were then treated, depending on the assay to be performed. Staining for α_2_β_1_integrin expression uses CD49b antibody, which detects the integrin α_2_ protein.

### Choice of tissues, LTP dose and post‐treatment time‐points in gene expression analysis

Primary culture LTP dose (3 min) and post‐treatment time‐points (0.5 and 2 h) for use in analysis of gene expression were optimised from previously obtained cell viability data 1 and preliminary testing on the RT^2^ Profiler Oxidative Stress qRT‐PCR Arrays. (Qiagen, Hilden, Germany) (Fig. S1). For each tissue pathology; Normal, BPH, Gleason 7 (G7) Cancer and Gleason 9 (G9) Cancer, three separate patient samples were used for the qRT‐PCR arrays. Details of all patient cultures used in the study are provided in Table S1. The G7 samples were selected as they were biopsied from patients where normal prostate tissue had also been removed, to give a ‘patient matched‐pair’.

### Dielectric barrier discharge jet configuration and cell treatments

The LTP jet consisted of a quartz glass tube of inner/outer diameter 4/6 mm, with two copper tape electrodes 20 mm apart. One electrode was powered (6 kV sinusoidal voltage at 30 kHz) and the other grounded. Helium, the carrier gas, flowed at two standard litres per minute and was fed with 0.3% molecular oxygen admixture. Cells were exposed to the LTP jet at a distance of 15 mm from the end of the bottom electrode for 180 s in 1.5 mL centrifuge tubes, suspended in 1.5 mL of respective media or treated directly in 6‐well or 12‐well plates (Sarstedt, Numbrecht, Germany). The distance between the end of the glass tube and the media surface was ~ 2 mm. Treatment times up to 600 s did not raise the surface temperature of culture media above 36.5 °C, measured using a thermocouple. The temperature and relative humidity of the laboratory were ~ 20 °C and ~ 25% respectively.

### RT^2^ profiler PCR oxidative stress arrays

Oxidative stress arrays were purchased from Qiagen. RNA was reverse transcribed to cDNA with the RT^2^ First Strand Synthesis kit (Qiagen). The cDNA was combined with SYBR Safe Mastermix (Qiagen) and aliquoted across the array plate. All array plate qRT‐PCR was performed using the C1000 Thermal Cycler and CFX96 Real‐Time System (BioRad, Hercules, CA. USA) under the RT^2^ Array qRT‐PCR protocol; –10 min, 39 cycles of 95 °C for 10 s, 60 °C for 1 min. Data was assimilated using the CFX Manager 2.0 (BioRad) and analysed using the Qiagen online Data Analysis Center (http://www.qiagen.com/gb/shop/genes-and-pathways/data-analysis-center-overview-page/). Gene expression scatterplots generated in the software were of the Log_10_ $2^{-\Delta \Delta C_{t}}$ values plotted against each other (treated/untreated), significant upregulation was defined as a ≥ 2‐fold change in expression. The Qiagen Oxidative stress response arrays were qRT‐PCR plates consisting of 84 wells containing gene‐specific primers to transcripts responsive to oxidative stress, five wells for house‐keeping genes (HPRT1, GAPDH, B2M, RPLP0, B‐ACT) for relative fold change quantification, PCR control wells in triplicate, reverse transcription control wells in triplicate and a single genomic DNA contamination control well. Notch signalling arrays were processed in the same way.

### SDS/PAGE and Western blotting

Cell lysates were either prepared from frozen pellets or cells were lysed in 6‐well plates following LTP treatment. Cell pellet lysates were prepared using CytoBuster™ Protein Extraction Reagent (Novagen, Burlington, MA, USA) with protease inhibitors (Roche, Burgess Hill, West Sussex, UK), cell debris removed by centrifugation (17 000 ***g***, 5 min) and protein content measured by Pierce™ Bicinchoninic acid Protein Assay Kit (Thermo Fisher Scientific, Waltham, MA, USA). 20 μg of protein was loaded per well with the appropriate amount of 4× SDS protein sample buffer (40% glycerol, 240 mm Tris‐HCl pH 6.8, 8% SDS, 0.04% bromophenol blue, 15% beta‐mercaptoethanol). In‐plate cell lysates were prepared using 4× SDS protein sample buffer. Samples were sonicated for 3 × 10 s using the Sanyo Soniprep 150 (MSE, London, UK) and incubated at 95 °C for 5 min. Forty microlitre of lysate was loaded per well. Protein samples were resolved on 10% acrylamide gels. Precision Plus Kaleidoscope Pre‐stained Protein Standards (BioRad) was used as marker for all gels. Proteins were transferred onto PVDF Immobilon P membranes (Merck Millipore, Burlington, MA, USA) for 1 h at 100 V. Primary and secondary antibodies and dilutions used in Table S2. Membranes developed in BM Chemi‐luminescence western blotting Substrate (POD; Roche) and viewed on the GeneGnome XRQ (Syngene, Cambridge, UK).

### Densitometry analysis

All western blot images were edited on GIMP 2.8 (GNU Image Manipulation Program, Berkeley, CA, USA) prior to densitometry analysis. Densitometry acquisition was performed using Image Studio Lite 5.2 (Licor, Lincoln, NE, USA). Readout from the densitometry acquisition allowed results to be analysed using Excel 2010 (Microsoft, Redmond, WA, USA). All graphs and statistics were prepared and performed on prism 7 (GraphPad, San Diego, CA, USA).

### Statistical analyses

All statistics performed on patient grouped gene expression were unpaired *t*‐tests (one‐tailed). Degrees of freedom were dependent on the *n* of the experiment which is supplied in the figure legends. *P ≤ 0.05, **P ≤ 0.01, ***P ≤ 0.001, and ****P ≤ 0.0001. The microarray data analysed by the Transcriptome Analysis Console program using ANOVA, values are provided in Table S3.

### RNA integrity and affymetrix clariom D microarray

RNA integrity testing was performed on all samples prior to microarray analysis, using the 2100 Bioanalyzer (Agilent, Santa Clara, CA, USA) and all samples returned a maximum RIN score of 10. Microarray analysis was performed by Eurofins using the Clariom D Microarray system (Affymetrix, Santa Clara, CA, USA).

### Bioinformatic analysis of microarray data

Initial analysis was performed using Gene Level Differential Expression Analysis on the Transcriptome Analysis Console Ver.3.1.0.5 (Affymetrix) equipped with the Clariom_D_Human.na36.hg.probeset. Pre‐processed data were analysed using the LIMMA (Linear Models for Microarray and RNA‐Seq Data) within the R numerical environment (Team RC. R: A language and environment for statistical computing. Foundation for Statistical Computing, Vienna, Austria ( https:///www.R_project.org/). Cell of origin and treatment type were modelled in the design matrix. Significant results after empirical Bayesian smoothing of the standard errors were extracted using a false discovery rate threshold of 0.025. No log‐fold change threshold was applied. After LIMMA analysis, results were analysed using gene set enrichment. The topGO package was used (Rahnenfuhrer AAaj. topGO: Enrichment Analysis for Gene Ontology. R package version 2.28.0 ed 2016). GO testing was performed using the ‘weight01’ algorithm with Fisher's statistics. A threshold of 0.05 was used to select significant results. As well as GO enrichment, pathway analysis was performed against the KEGG 11 pathways. Significant results from the LIMMA analysis were analysed with a *P*‐value threshold of 0.05.

### Quantitative reverse transcription PCR (qRT‐PCR) – Cell line and array validation

All RNA extractions (both for individual qRT‐PCRs and for the RT^2^ Profiler qRT‐PCR Arrays) were performed on cell pellets previously stored at −80 °C or by direct in‐plate lysis (addition of complete RLT buffer followed by storage at −80 °C overnight) using the RNeasy Micro Kit (Qiagen) following the manufacturer's instructions for the animal cell protocol. Genomic DNA contamination was removed by on‐column application of the RNase‐free DNase Set (Qiagen). cDNA was synthesised using Superscript III cDNA synthesis kit (Invitrogen, Carlsbad, CA, USA), with reactions placed in GeneAmp PCR System 9700 thermal cycler (Applied Biosystems, Foster City, CA, USA). Taqman qRT‐PCR was then performed in FrameStar 96 qRT‐PCR plates (4titude, Dorking, UK). Input cDNA was standardised at 30 ng per well. The mastermix used was TaqMan Fast Universal Master Mix (Applied Biosystems). Probes used in experiments are provided in Table S4. qRT‐PCR was performed using the C1000 Thermal Cycler and CFX96 Real‐Time System (BioRad) under the following protocol; 95 °C – 10 min, 39 cycles; 95 °C for 10 s, 60 °C for 1 min, 4 °C Hold. Data was assimilated using the CFX Manager 2.0 (BioRad) and analysed using the $2^{-\Delta \Delta C_{t}}$ method, normalising to 18S. All boxplots and statistics were prepared and performed on prism 7 (GraphPad).

### Immunofluorescence

Cultured primary cells were deposited into BioCoat Collagen I 8‐well chamber slides (Corning) – 10 000 cells per well. An LTP dose of 1.5 min was administered directly to the well and cells were fixed 30 min after treatment with 4% paraformaldehyde. The reduced dose was used due to the smaller liquid volume of the well to limit cell death following treatment. Cells were permeabilised (if required) for 10 min in 0.5% Triton X‐100 (Sigma) in PBS. Cells were blocked in 10% goat serum in PBS for 1 h. Primary antibody was diluted in 10% goat serum in PBS. For antibodies and dilutions used see Table S2. The chamber slides were left overnight at 4 °C on SSM3 orbital shaker (Stuart, Staffordshire, UK) at 50 r.p.m. After overnight incubation, a secondary Alexafluor 568 antibody ‐ anti‐rabbit A11036 (Invitrogen), anti‐mouse A11031 (Invitrogen) ‐diluted 1 : 1000 in 10% goat serum was then applied for 1 h at room temperature. The chamber of the slides was then removed and Vectashield with DAPI (Vector, Peterborough, UK) was applied to the slide with a coverslip applied on top before being sealed. Slides were imaged on the DM IL LED Microscope (Leica, Wetzlar, Germany) with the DFC365 FX Camera (Leica) under Cy3 and DAPI filters. Images were viewed and stored using the LAS X program (Leica).

### Colony forming assay

Cultured primary prostate epithelial cells were plated and treated with 10 μm RO4929097 or 0.1% DMSO for 3 days and then exposed to 2 Gy radiation. Cells were selected and plated (×3) at colony forming density and allowed to grow. After 7–10 days colonies were stained with crystal violet (1% crystal violet/10% ethanol/89% PBS) and counted.

### *In vivo* experiments

Patient‐derived xenografts were grown in Rag2−/−gamma(C)−/− mice and were harvested at appropriate size. Cells were then disaggregated and depleted for mouse cells, counted and then treated for 24 h with 10 μm RO4929097 or 0.1% DMSO, *ex vivo*. Following incubation, cells were injected (10^3^ or 10^4^ cells) with 2 × 10^5^ STO feeder cells and 80 μL matrigel, subcutaneously. Tumour volume was measured.

## Results

### LTP induces an oxidative stress response in primary prostate basal epithelial cultures

To assess the cellular reaction to LTP, we assembled a panel of primary prostate epithelial cultures from normal (*n* = 3), benign (*n* = 3) and malignant tissue [Gleason 7 (G7) (*n* = 3) and Gleason 9 (G9)(*n* = 3)] and analysed the expression of 84 genes at 2 h following a 3‐min LTP dose, using Qiagen Oxidative Stress Profiler Arrays (Fig. 1B–E). The time and dose were chosen after optimisation experiments (Fig. S1), as there is a very rapid and transient upregulation of the anti‐oxidative responses. The normal and G7 cultures originated from three patients and were defined as ‘patient matched‐pairs’. This afforded a true comparison between normal and cancer gene transcription (Table S1).

The transcriptional signature after LTP treatment indicated rapid upregulation of (a) redox enzymes (HMOX1, SRXN1, TXNRD1), (b) chaperones involved in protein folding and autophagy (SQSTM1, HSPA1A) and (c) a MAPK phosphatase (DUSP1) (validation in Fig. S2). HMOX1 and HSPA1A were upregulated in all pathology types. The other genes were upregulated in at least two tissue pathologies, except for DUOX1 and GPX4, which were exclusively responsive in a single Gleason 9 sample. Since these genes were upregulated across all, or several, tissue pathologies, we next sought to identify common upstream transcription factors that could govern the LTP response of the prostate epithelial cells.

### Oxidative stress transcription factors are activated in LTP‐treated primary cultures

**Figure 2:**
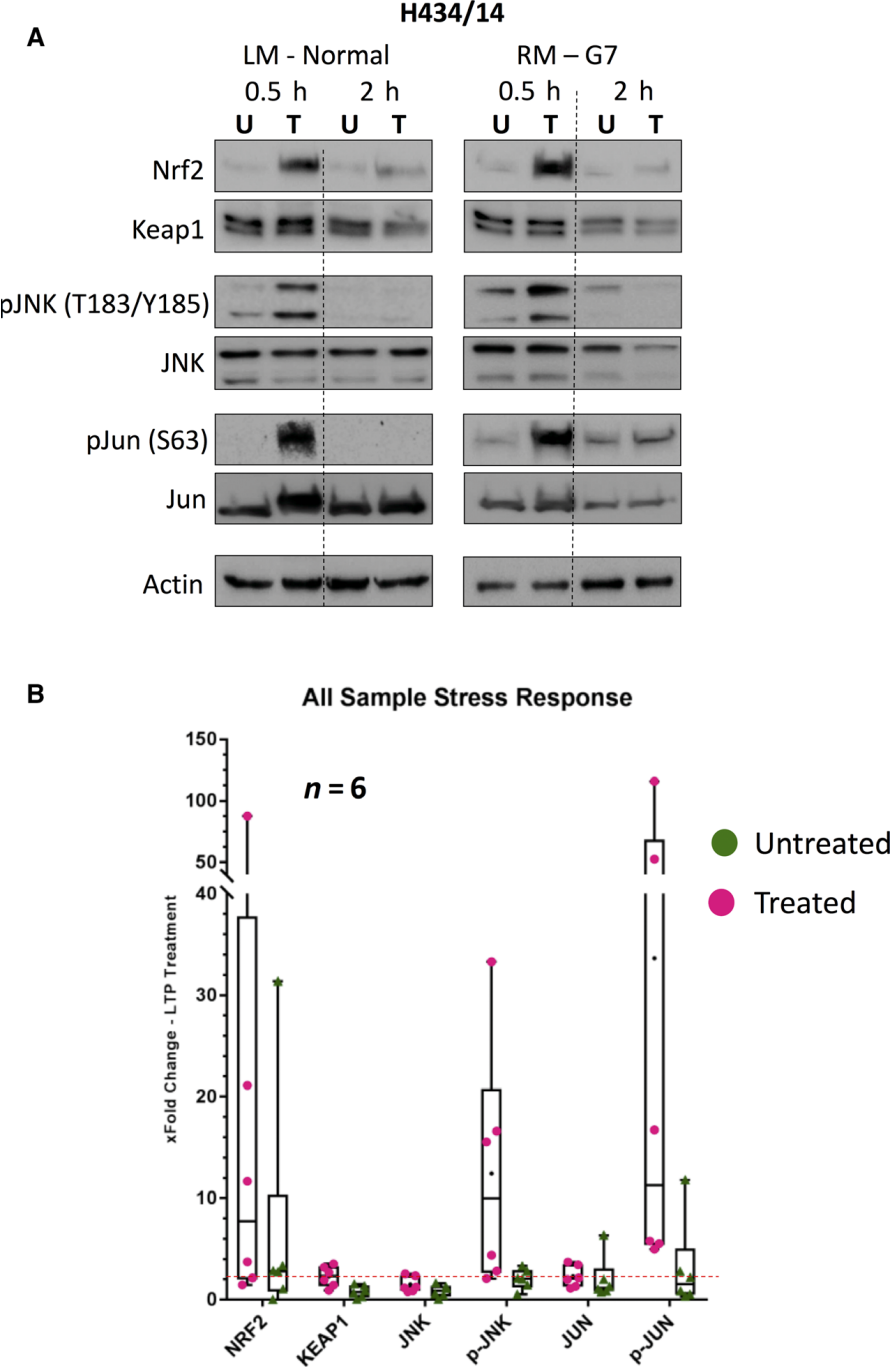
LTP causes accumulation of Nrf2 and activation of AP‐1. (A) Western blot analysis of stress activated transcription factors in untreated (U) and treated (T) cells, 0.5 and 2 h after treatment. Three matched pairs were tested with one example represented here and two further examples in Fig. S5. (B) Boxplot densitometry analysis of the protein data. All six patient samples (normal *n* = 3, cancer *n* = 3) are plotted as single points and mean fold change represented by (−). The red dotted line represents a 2‐fold change.

Previous studies have implicated activation of Nrf2, the canonical oxidative stress transcription factor, and AP‐1, a regulator that balances cell growth and death, in LTP response 2, 12, 13, 14. Protein analysis of three patient matched‐pairs indicated that, within 30 min of treatment, LTP stimulates both an accumulation of Nrf2 and activation of the AP‐1 pathway, through characteristic phosphorylation of both JNK and Jun (Figs 2 and S5). Protein levels of Keap1 remained constant after treatment, indicating that Nrf2 accumulation is due to cellular redox changes, rather than a manipulation in the amount of its negative regulator.

### Cellular stress pathways are activated by LTP

**Figure 3:**
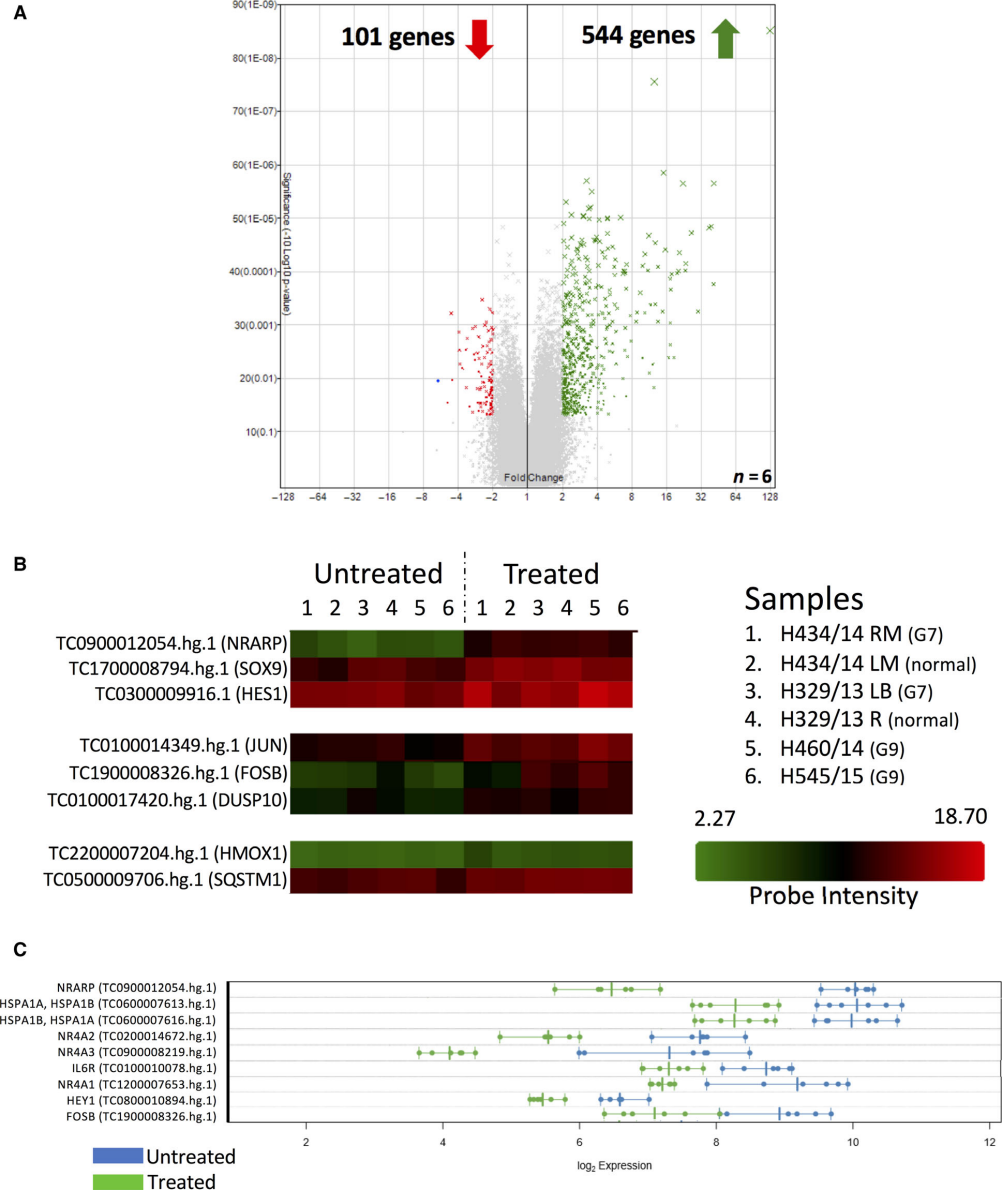
Whole transcriptome analysis reveals activation of multiple signalling pathways by LTP. Microarray analysis of gene expression 2 h after 3 min LTP dose in primary prostate cultures. Patient samples were grown in culture from normal prostate (two samples), Gleason 7 (two samples) and Gleason 9 cancer (two samples). Gene expression was assessed 2 h after 3‐min LTP dose. (A) Volcano plot of significantly changed gene expression (*P* =≤ 0.05, fold change cut‐off is ≥ 2‐fold) in Treated versus Untreated samples. 645 transcripts were altered by LTP – 544 upregulated, 101 – downregulated. (B) Heat map of genes of interest across the six samples tested. (C) Expression plot showing genes that passed LIMMA.

The oxidative stress PCR arrays clearly present a bias by nature of design, restricted to identifying only oxidative stress response post‐LTP. Therefore, whole transcriptome analysis of 6 primary samples (2× patient matched pairs and 2× Gleason 9) using microarrays was performed to assess the impact of LTP on global gene expression (Fig. 3). The patient‐matched pairs didn't exhibit a divergent gene signature after treatment, (Fig. S3) so all samples were grouped for further analysis and not segregated by disease status. Overall, 544 transcripts were significantly upregulated and 101 genes were significantly downregulated (fold change ≥ 2, *P* ≤ 0.05, relative to untreated) between the six samples (Fig. 3A). The microarray confirmed the oxidative stress gene upregulation, with HSPA1A, HMOX1 and SQSTM1 all significantly upregulated in LTP treated samples (Fig. 3B). From initial observation (Fig. 3B and Table S3) and after LIMMA (Linear Models for Microarray and RNA‐Seq Data) analysis (Fig. 3C and Table S5), which accounts for a gene's inherent biological expressional variability, several signalling pathways were activated by LTP.

A striking activation of Notch target genes (e.g. NRARP, HES1, HEY1 etc.) was detected. Indeed, the top hit from the entire microarray was NRARP, a target and downstream effector of Notch, and a negative regulator of cleaved Notch (Fig. 3B,C). Expression of 9 genes was assessed by qRT‐PCR in four primary cultures to validate the microarray data‐set (Fig. S4). These genes were chosen to represent Notch (NRARP, SOX9, HES1), AP‐1 (JUN, FOSB, DUSP10) and Nrf2 (HMOX1, SQSTM1) stress responses. Whilst some of these genes failed to achieve ≥ 2‐fold upregulation cut off in all samples in the qRT‐PCR, the overall validation was successful in confirming the robustness of the microarray dataset.

### AP‐1 signalling and Notch1 signalling is activated by LTP in prostate basal epithelial cells

**Figure 4:**
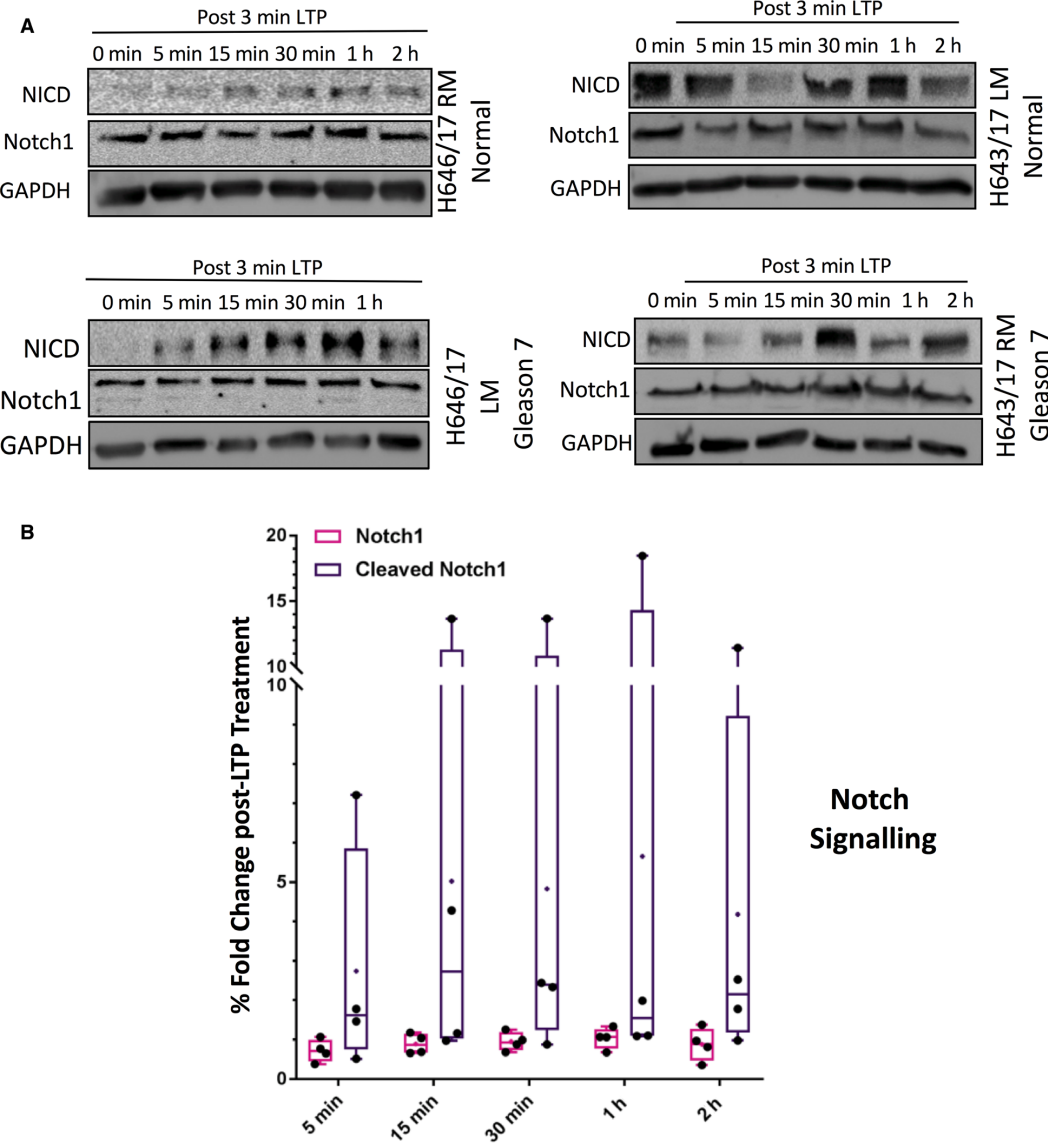
LTP activates Notch signalling in primary prostate epithelial cell cultures. (A) Western blot time‐course of protein alterations in Notch signalling pathways following 3 min plasma treatment in two matched pairs of normal and cancer biopsies (one shown here and one in Fig. S7). (B) Densitometry analysis of Notch1 activation by LTP in all four samples.

The upstream components of the signalling pathways identified as responding to LTP in the microarray were assessed by Western Blot, through observation of protein changes over a 2‐h time‐course in two matched pairs. We monitored AP‐1 transcription factor further by assessing levels of Jun and P‐Jun, one of the subunits of the AP‐1 transcription factor (Fig. S6). We monitored activation of Notch by assessing levels of Notch and levels of NICD (Notch intracellular domain), which is cleaved, active Notch (Fig. 4). Total levels of Notch1 were unchanged by treatment. However, NICD, the active cleaved form of Notch, accumulated in treated cells to ~ 5‐fold greater levels than in untreated cultures half an hour after LTP dose, though there was wide variation between the samples.

### Endogenous Notch gene expression and activation of Notch signalling in response to LTP are both enhanced in the progenitor cell population

**Figure 5:**
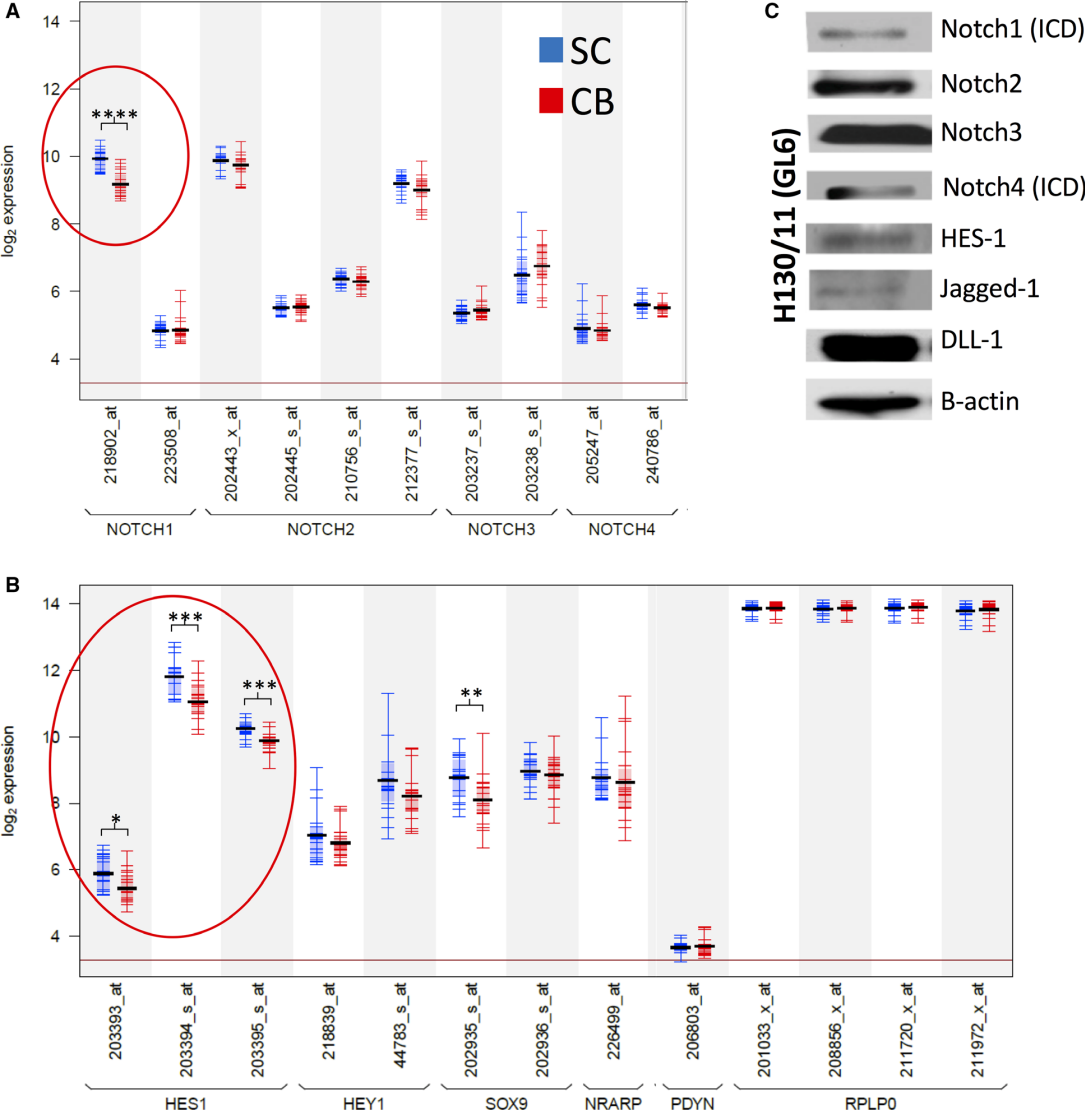
Protein and RNA expression of Notch in primary prostate epithelial cell cultures. (A) and (B) – Expression of Notch receptors and target genes in primary prostate epithelial SC and CB cells. Silenced gene PDYN and Active gene RPLP0 are provided as negative and positive expression controls respectively. (Data obtained from Birnie *et al*. 16), (Significance; *P ≤ 0.05, **P ≤ 0.01, ***P ≤ 0.001, ****P ≤ 0.0001). (C) Expression of Notch receptors and target proteins in primary prostate epithelial cells (one patient culture shown).

**Figure 6:**
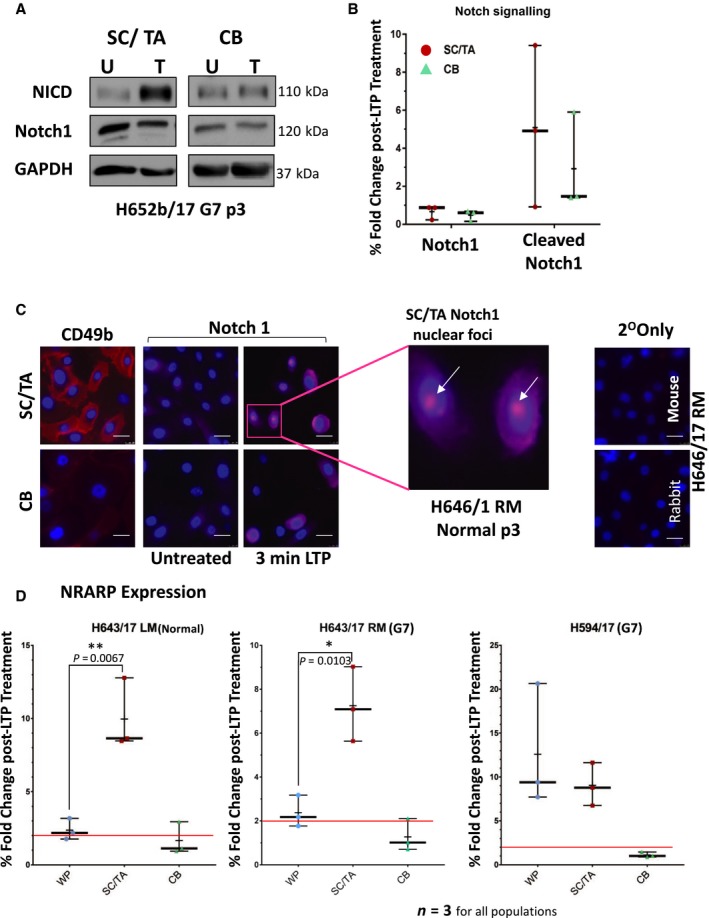
Notch signalling is more active in the SC/TA population, than in CB cells, after LTP treatment. (A), (B) Western blot and densitometry analysis of Notch1 activation in prostate epithelial sub‐populations 30 min after 3 min LTP dose (one sample shown, two more samples shown in Fig. S8). (C) Immunofluorescence images of Notch1 in prostate epithelial sub‐population cells 30 min after 3 min LTP dose. Validation of cellular fractionation is shown by the CD49b staining of SC/TA. Nuclear foci in SC/TA population highlighted in expanded box (white arrows). (D) Gene expression boxplots of sub‐population NRARP expression 2 h after 3 min LTP dose. Each biological repeat (*n* = 3) is plotted as a point and the mean expression value is represented as a (−). The red line shows a 2‐fold change in expression. Unpaired *t*‐tests (one‐tailed) were performed between each subpopulation. White scale bars = 25 μm. (*P < 0.05, **P < 0.01).

Active Notch signalling is associated with epithelial SC pools and is a determining factor in cellular identity 15. NOTCH1 and HES1 are expressed at high levels in the SCs of primary prostate basal epithelial cultures, in comparison to more differentiated CB cells, and the receptor has also been implicated as a key determinant of the human basal SC population 16, 17 (Fig. 5). Preliminary protein analysis of Notch1 signalling in the selected basal epithelial cell populations (SC/TA and CB) revealed activation after LTP treatment (Fig. 6A,B) with the predominant activation being seen in the SC/TA population. Immunofluorescence indicated an increase in Notch post‐LTP treatment, but nuclear staining of the active NICD was only observed in treated SC/TA cells (Figs 6C and S7). Preferential activation of Notch in the progenitor cells was further confirmed by upregulation of NRARP following LTP, where all three cultures exhibited significantly higher responsive expression of the negative regulator in their SC/TA population over that observed in CB cells (Fig. 6D). Jun was phosphorylated in all primary cultures exposed to LTP (peak intensity at 1 h) following treatment but was not cell‐specific (Fig. S9).

### Inhibition of Notch using gamma‐secretase inhibitors induces cell differentiation and reduces colony forming efficiency

**Figure 7:**
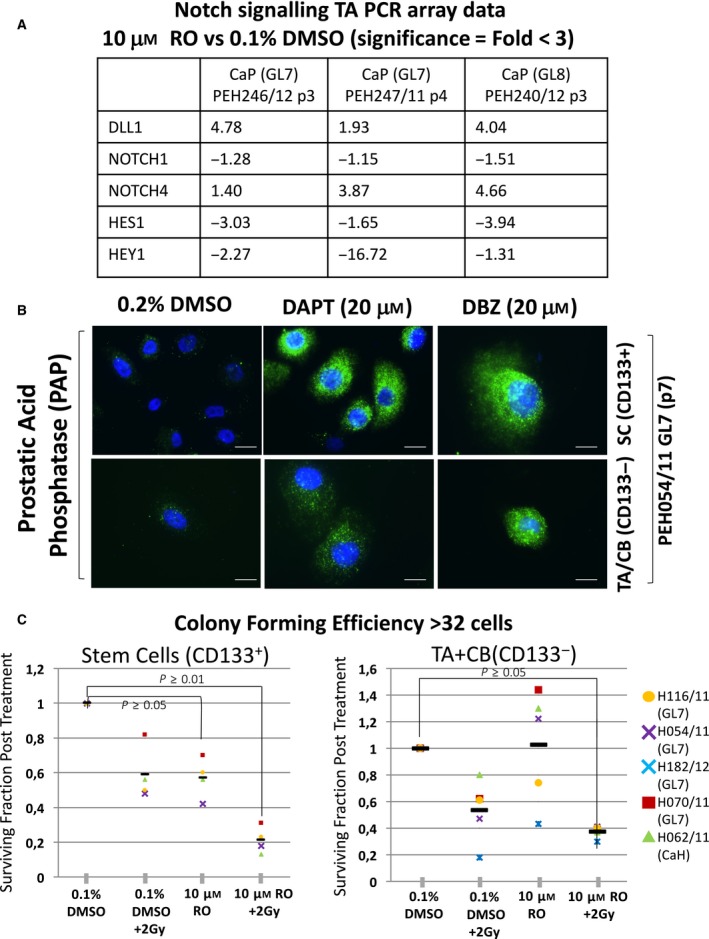
Exposure of primary prostate epithelial cells to a gamma‐secretase inhibitor results in Notch inhibition, cell differentiation and a reduced colony forming ability (A) Primary prostate epithelial cell cultures were treated with 10 μm RO and the gene expression assessed using Qiagen Notch Signalling PCR Array. Shown are the fold changes of a selection of Notch1 signalling genes. (B) Inhibition of Notch using two different gamma‐secretase inhibitors (DAPT, DBZ) results in increased prostatic acid phosphase (PAP) protein expression. (C) Colony forming efficiency of CD133^+^ and CD133^−^ prostate epithelial cell fractions treated with gamma secretase inhibitors alone or with radiation (2 Gy) (*n* = 5). White scale bars = 25 μm.

Notch inhibition can be achieved by addition of gamma‐secretase inhibitors, which prevent cleavage of Notch to its active form. After addition of RO inhibitors RO (RO4929097), DAPT and Dibenzazepine (DBZ), assessment of gene expression was carried out using a Qiagen Notch Signalling PCR Array. The data confirmed inhibition of Notch1, and a coincident decrease in HES1 and HEY1, which are downstream Notch signalling transcription factors (Fig. 7A). There was however a compensatory increase in Notch4 gene expression and upregulation of DLL1, which is a ligand that binds to the extracellular domain of the Notch receptor. As a result of Notch inhibition, we observed an induction of cellular differentiation in the primary prostate epithelial cell cultures, as shown by an increase in PAP expression (Fig. 7B). Functionally, this inhibition of Notch resulted in a decrease in colony forming efficiency of 30–60% in SC. The effects of Notch inhibition in TA/CB cells gave a wide variation in response in different patients. In both SCs and TA/CB cells, the application of 2 Gy radiation, in combination with Notch inhibition resulted in strikingly reduced colony forming ability (> 70% reduction in SCs and > 60% reduction in TA/CB cells (Fig. 7C).

### Inhibition of Notch in a patient‐derived xenograft reduces both tumour‐initiating ability and tumour growth

**Figure 8:**
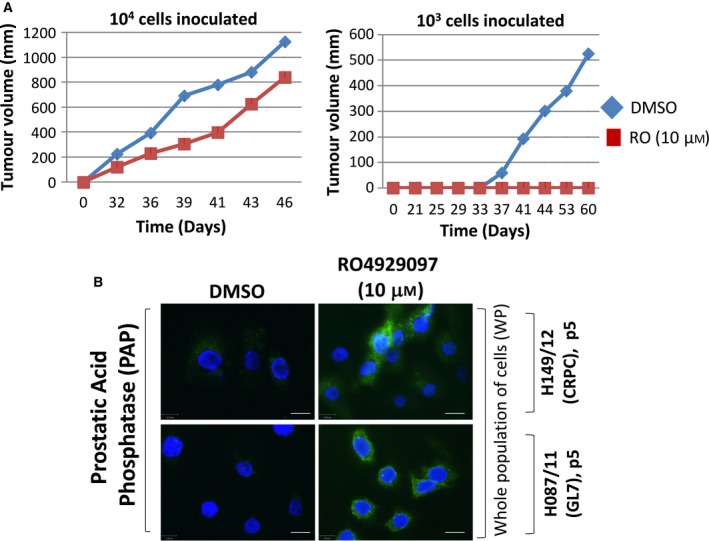
Use of gamma‐secretase inhibitor to treat patient‐derived xenograft cells results in reduced or (A) Tumour growth *in vivo* of cells derived from patient‐derived xenografts after *ex vivo* treatment (24 h) with and without gamma‐secretase treatment. (B) PAP immunofluorescence in human cells extracted from patient‐derived xenograft tumours, with and without gamma‐secretase inhibitor treatment. White scale bars = 25 μm.

In order to assess the effect of Notch inhibition on tumour growth *in vivo*, disaggregated cells from a patient‐derived xenograft were treated *in vitro,* then re‐injected back *in vivo*. Effects on tumour initiation and growth post‐treatment were both monitored (Fig. 8A). Depending on the number of cells used in the injection, tumour growth was either prevented, delayed or reduced. In addition, once the tumours had been collected and disaggregated with mouse cells removed, the remaining human cells were stained for an indicator of cell differentiation, PAP, and it was found that all tumours which grew post‐Notch inhibition had increased expression of PAP (Fig. 8B).

## Discussion

This is the first report of global gene expression analysis of LTP in multiple patient‐derived primary epithelial prostate cells, including matched pairs of normal and cancerous prostate from the same patient. Induction of oxidative stress, with typical accumulation of the antioxidant master regulator, Nrf2 and activation of AP‐1 transcription factor was the primary and predictable observation, but nonetheless novel because of the cell model that we used. LTP‐induced oxidative stress has previously been observed in cancer cell lines 1, 18. Differences in base Nrf2 levels and the kinetics of Jun phosphorylation, as an indicator for AP‐1 signalling, were likely due to the innate variability in the oxidative stress buffering capacity of normal and cancerous epithelia and also the different patient origins of the tissue, all of which will have variable levels of antioxidant and ROS scavenging proteins. To our knowledge, this study represents the only true normal‐cancer comparison of tissue isolated from the same patient to date, with no artificial immortalisation or culture media differences to add further variables and artefacts upon analysis.

The ‘selective effect’ of cancer cells showing more sensitivity to LTP treatment than their normal counterparts has been a contentious claim in the field of anti‐cancer plasma treatment, and significantly our study showed no such ‘selective effect’. Studies that arrive at this conclusion either used normal cells from different organs (to the tumour cell origin) 19, 20, 21, 22 or compared epithelial with mesenchymal cells 13. When normal and cancer from the same tissue and cellular origin have been compared 20, inter‐patient variance and the imposed immortalisation of the ‘normal’ cell line have not been considered.

In the global gene expression analysis of LTP‐treatment in patient‐derived cancer cells, activation of Notch signalling was identified as a novel pathway. We have shown for the first time that LTP activates Notch signalling, with (a) stimulation of downstream genes, (b) cleavage of the receptor to release the active NICD and (c) nuclear localisation of the transcription factor exclusively in the progenitor cell fraction of primary prostate epithelial cell cultures. Notch signalling is important in prostate organogenesis 23, development, differentiation, and tumourigenesis (reviewed in 24). The pathway regulates self‐renewal (asymmetric division) and prevents differentiation in normal and cancer SC populations 15, 25, 26, 27. Notch signalling also defines the self‐renewal capacity of bipotent basal SCs in the human prostate 17. Both Notch and Notch ligand‐receptor proteins are expressed in primary cultures (Fig. 5). Application of gamma‐secretase inhibitors to prevent cleavage of the NICD and activation of Notch signalling, with and without ionising radiation, reduced the self‐renewal capabilities of prostate CSCs, both *in vitro* (Fig. 7C) and *in vivo* (Fig. 8) by forcing differentiation (Figs 7B and 8B). The observed variations in colony forming efficiency in TA/CB cells from different patient samples is probably the result of different ratios of TA:CB cells in individual patient‐derived cultures, and it would be predicted that the Notch inhibitor would have more effect on the TA cells than the CB cells. This data suggests that Notch signalling is critical for prostate SC maintenance, both *in vitro* and *in vivo*, as pharmacological inhibition of pathway activation results in a reduction of self‐renewal capabilities and pushes epithelial cell differentiation towards a luminal phenotype.

Low Temperature Plasma treatment induced upregulation of the negative regulator; NRARP, a Notch‐specific target and downstream effector 28 that promotes Notch degradation leading to release of NCID 29. This was confirmed by observation that other canonical Notch targets were also LTP‐responsive, including HES1 and HEY1, which are transcriptional repressor proteins, acting to maintain cells in an undifferentiated state, by limiting lineage specific transcription factor expression 30. Constitutive expression of active Notch1 in embryonic SCs 31 identified a transcriptional signature strikingly similar to that of the LTP treated patient cultures. Convergent significantly upregulated hits included; NRARP, HEY1, HES1, BTG2, GADD45B, SOX9, RHOV, EFNA1, HBEGF, ITPKC, RIPK4, ATF3 and the EGR factors, supplying correlative evidence that Notch signalling is active, and acting as a master regulator, in LTP‐treated prostate progenitor cells.

Notch signalling has also emerged recently as a modulator of oxidative stress. Exogenous ROS are able to upregulate cyto‐protective Notch1 signalling with associated downstream gene expression in mesenchymal SCs 32. The NICD itself can act as a potent inhibitor of cell death signals in oxidative stress conditions 33. Crosstalk between Notch and other pathways, such as NF‐kB 34, 35, 36 and Nrf2 37 has been observed, to which some of the transcription factor's pleiotropic effects may be attributed.

The activation of stress‐activated protein kinases, by excess ROS, can force SC differentiation 38, 39, a fate that can be averted by Notch signalling 40. In the primary cultures following LTP treatment, we observed both phosphorylation of JNK and subsequent activation of the Notch receptor. This is accompanied by a decrease in colony forming efficiency, indicative of a reduction in self‐renewal capability 1, and thus depletion (by symmetrical division), of SCs that fail to trigger Notch in high ROS conditions. We have previously observed that a pool of prostate epithelial cells survive LTP 1. By activating Notch, the resistant subpopulation could prevent depletion of the progenitor cells by ROS‐induced differentiation (Fig. 5B).

Furthermore, as a result of stimulation of Notch signalling by radiation, another ROS‐inducing therapy, we have shown that Notch inhibition promotes differentiation and reduces colony forming ability. Indeed, Notch signalling has been identified as a radiation resistance mechanism in various cancers, and Notch inhibition has therefore been proposed as a possible therapeutic to be included with radiotherapy 41. More specifically, Notch has been identified as part of a radiation resistance mechanism particularly within cancer SCs 42, 43. Our study concurs with this idea, i.e. that the progenitor cells respond differently to the LTP treatment, and with increased Notch signalling could be more resistant to ROS‐inducing therapies than more differentiated cells.

**Figure 9:**
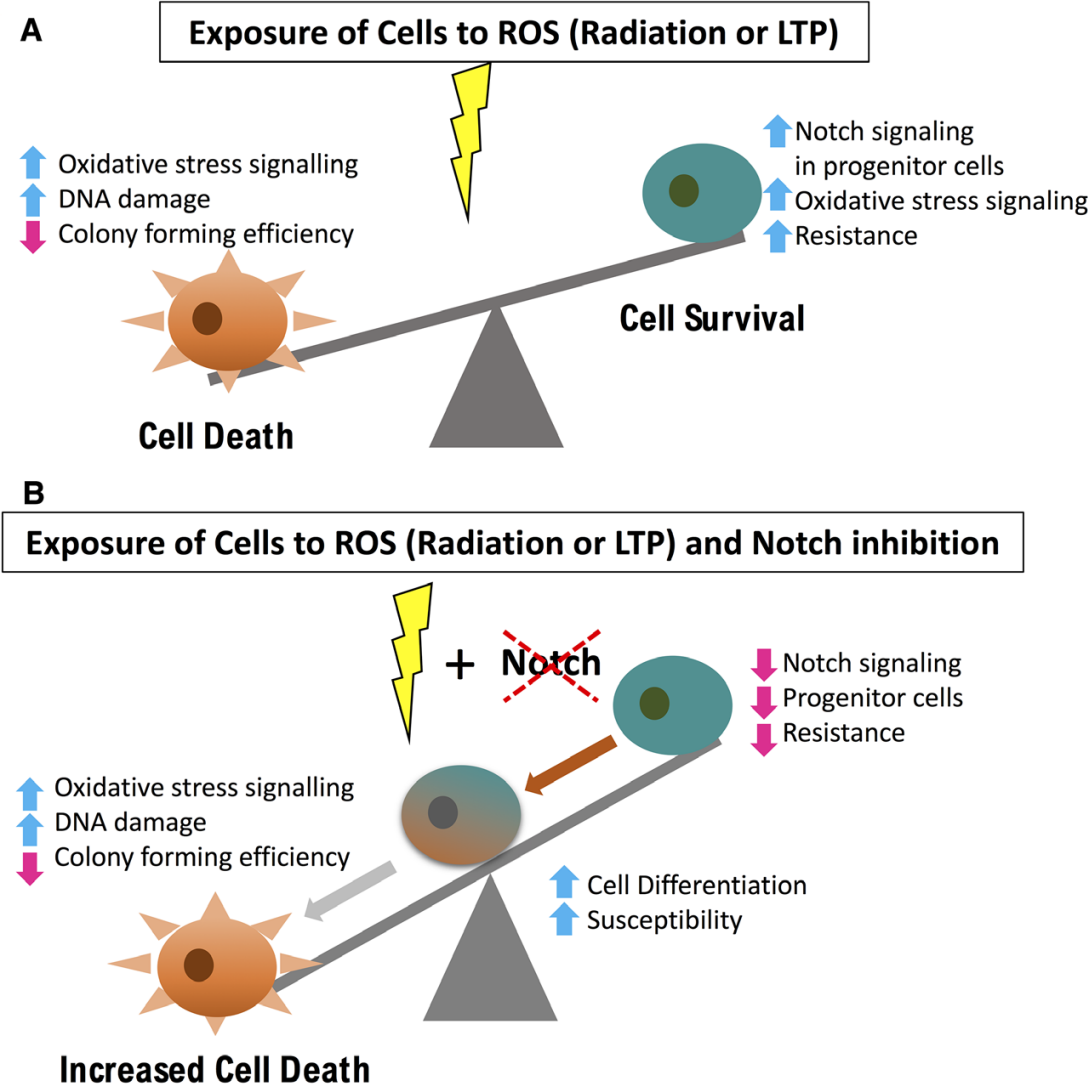
Proposed model for mechanism of action of Notch Inhibitors in Combination with ROS‐inducing Treatments. There is a heterogeneous cell response to ROS‐inducing treatments (Radiation or LTP). (A) Some cells will succumb to increased ROS, DNA damage and cell death. Other cells, typically early progenitor cells have Notch signaling as a resistance mechanism resulting in maintenance of SC phenotype, resistance to treatment and cell survival. (B) Our results propose that a combination of Notch inhibition with ROS‐inducing treatment results in reduced Notch signaling and progenitor cell differentiation thus leading to increased susceptibility, reduced resistance and increased cell death.

Taken together, we propose a model that postulates a Notch inhibition/ROS‐inducing treatment would be more effective in prostate cancer therapies than either treatment alone (Fig. 9). The cell heterogeneity of prostate tumours, as modelled here by patient‐derived primary epithelial prostate cell cultures means that there is a heterogeneous response to ROS‐inducing stimuli, including an increased stress response, DNA damage and cell death in more differentiated cells, and increased Notch signalling, resistance mechanisms and cell survival in progenitor cells (Fig. 9A). If a combination therapy is to be used, combining Notch inhibition with a ROS‐inducing therapy, then decreased Notch signalling should result in cell differentiation, reduced resistance and increased susceptibility leading to increased cell death (Fig. 9B).

The importance of Notch signalling in prostate cancer is not only significant when considering ROS‐inducing therapies like radiation and LTP, as shown here, but is also of importance when considering androgen‐deprivation therapy (ADT). Although ADT can work initially for most people, castration‐resistant cancer is almost inevitable 44, 45. Therefore, elucidating the mechanisms of resistance to ADT is necessary. Three recent studies have shown that Notch signalling can contribute to resistance to enzalutamide (androgen receptor antagonist) 46 and that Notch inhibition combined with ADT can cause a synergistic therapeutic effect 47, 48. In addition, Notch signalling has also been implicated in docetaxel resistance 49. Therefore, Notch inhibitors are a significant candidate to consider in a variety of combination therapies, particularly in prostate cancer 50. However, despite approvals for clinical use before implementation, the regulation of Notch signalling in all cell types within a heterogeneous prostate tumour would need to be elucidated further since Notch is involved in many complex cellular processes both in cancer and in normal tissue, and has been implicated as both an oncogene and tumour suppressor 51.

## Author contributions

JRP carried out the majority of experiments, analysed the data and prepared the manuscript. AMH provided expert advice and training to JRP on the low temperature plasma device. APD performed bioinformatics analysis. RA performed notch inhibitor experiments (colony forming/staining/*in vivo*). MSS, an urological surgeon, provided samples obtained from patients. VMM, a tissue procurement officer, retrieved and couriered samples, and organised patient information database. FMF conceived initial PCR array experiments, analysed some data and retrieved information for manuscript preparation. DO and NJM supervised JRP, prepared the manuscript, performed data analysis discussion and obtained funding for the study.

## Supporting information


**Fig. S1.** Optimisation of post‐treatment timepoint and LTP dose on the oxidative stress profiler arrays.

**Fig. S2.** Results of qRT‐PCR arrays are reproducible and valid.

**Fig. S3.** Normal and Gleason 7 culture transcriptional response to LTP is similar.

**Fig. S4.** Gene expression analysis by qRT‐PCR validates microarray results.

**Fig. S5.** Low Temperature Plasma causes accumulation of Nrf2 and activation of AP‐1.

**Fig. S6.** Low Temperature Plasma activates AP‐1 signalling in primary prostate epithelial cell cultures.

**Fig. S7.** Notch signalling is more active in the SC/TA population, than in CB cells, after LTP treatment.

**Fig. S8.** AP‐1 signalling is active in both the SC/TA cell population and the CB cell population after LTP treatment.

**Fig. S9.** AP‐1 signalling is active in primary prostate epithelial cell cultures after LTP treatment.

**Table S1.** Patient information of all cell cultures used in the study.

**Table S2.** Antibodies used in the study.

**Table S3.** All samples ‘treated against untreated’ Excel file.

**Table S4.** Taqman probes used in the study.

**Table S5.** LIMMA meta‐analysis of ALL samples ‘treated against untreated'’ Excel file.

## Data Availability

Research data pertaining to this article is located at https://www.ncbi.nlm.nih.gov/geo/query/acc.cgi?acc=GSE119052
