# Supplementary figures and images for: Notch signalling is a potential resistance mechanism of progenitor cells within patient‐derived prostate cultures following ROS‐inducing treatments
Source: FEBS Lett. 2019 Sep 17;594(2):209–26. doi: 10.1002/1873-3468.13589 (PMC7003772; doi:10.1002/1873-3468.13589)

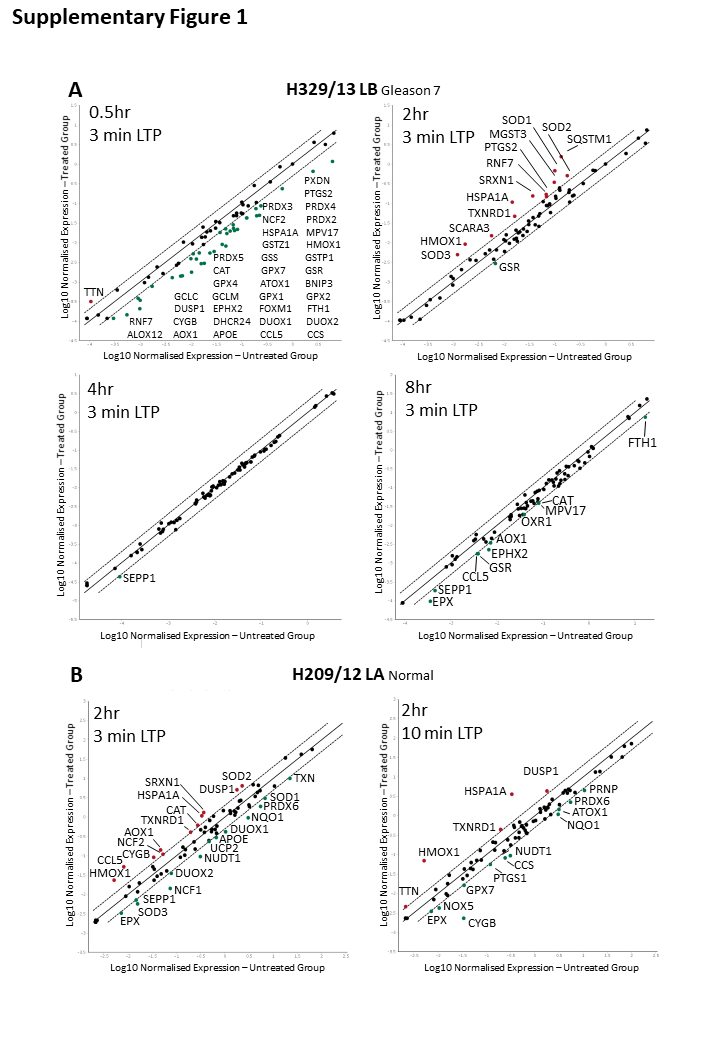

Supplement: Supplementary file 1 — Fig. S1. Optimisation of post‐treatment timepoint and LTP dose on the oxidative stress profiler arrays. [file FEB2-594-209-s001.tif]

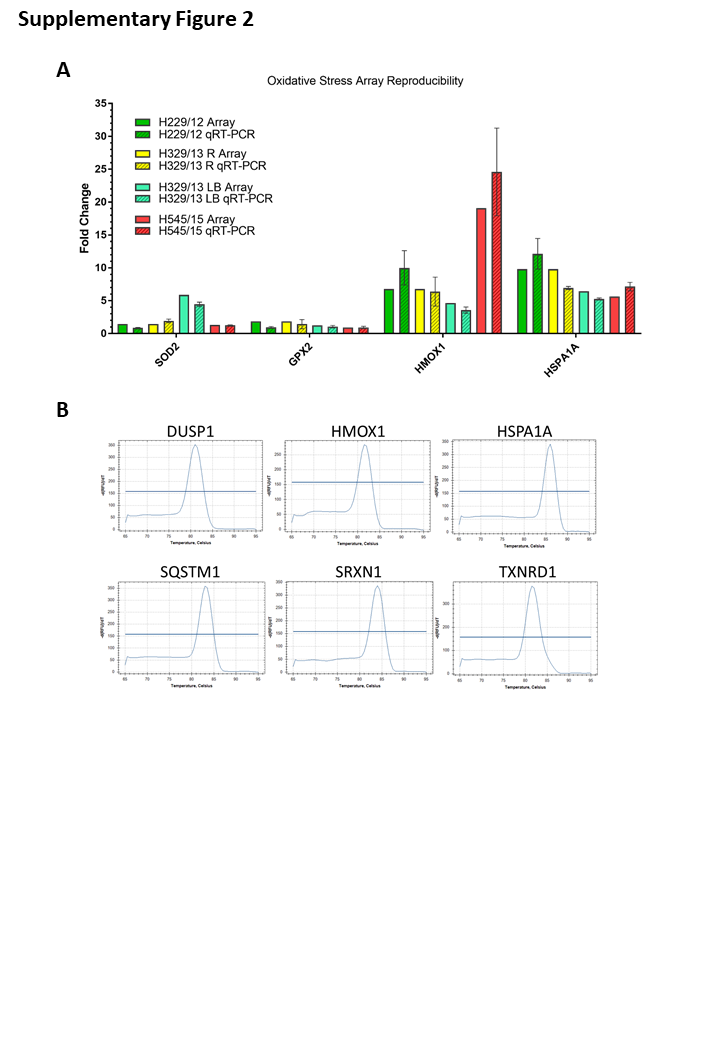

Supplement: Supplementary file 2 — Fig. S2. Results of qRT‐PCR arrays are reproducible and valid. [file FEB2-594-209-s002.tif]

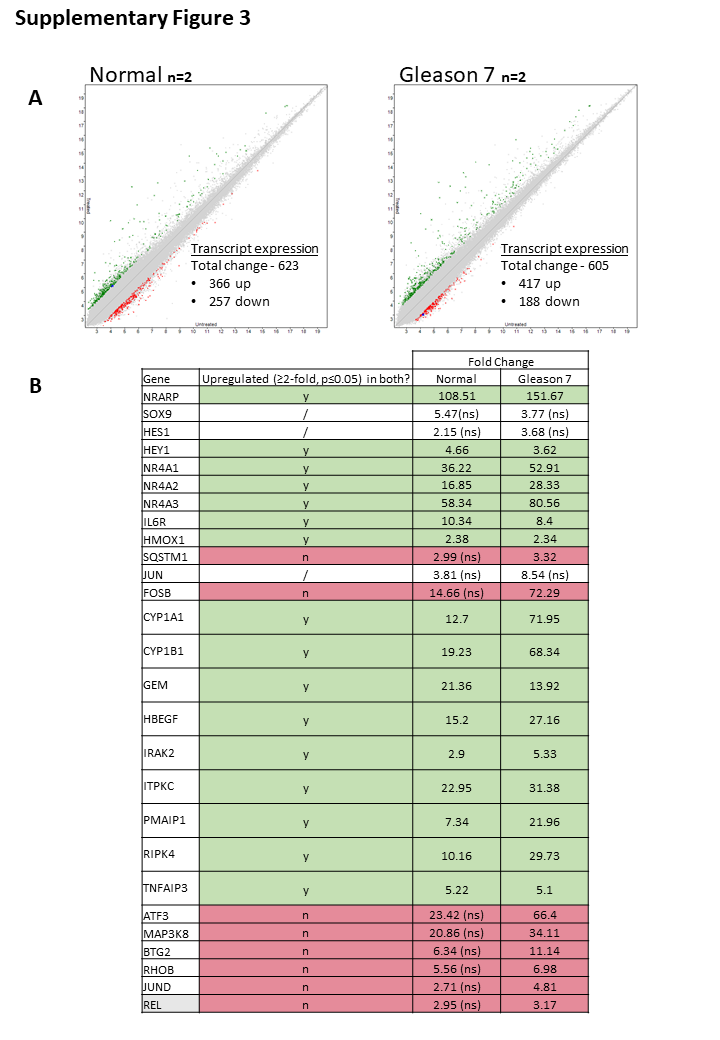

Supplement: Supplementary file 3 — Fig. S3. Normal and Gleason 7 culture transcriptional response to LTP is similar. [file FEB2-594-209-s003.tif]

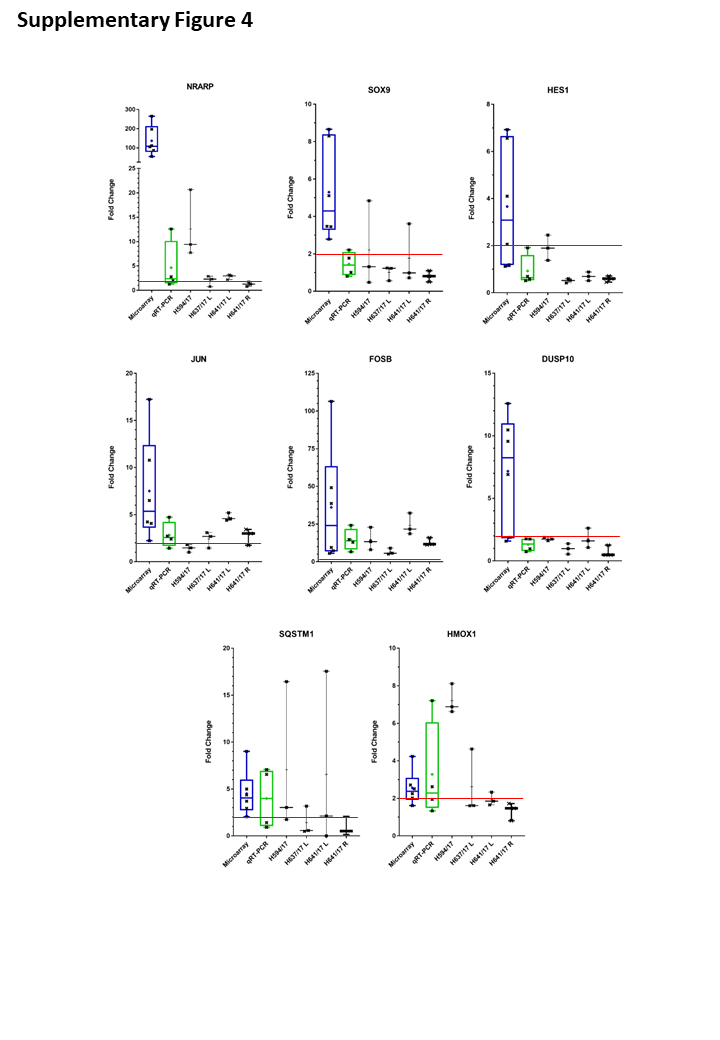

Supplement: Supplementary file 4 — Fig. S4. Gene expression analysis by qRT‐PCR validates microarray results. [file FEB2-594-209-s004.tif]

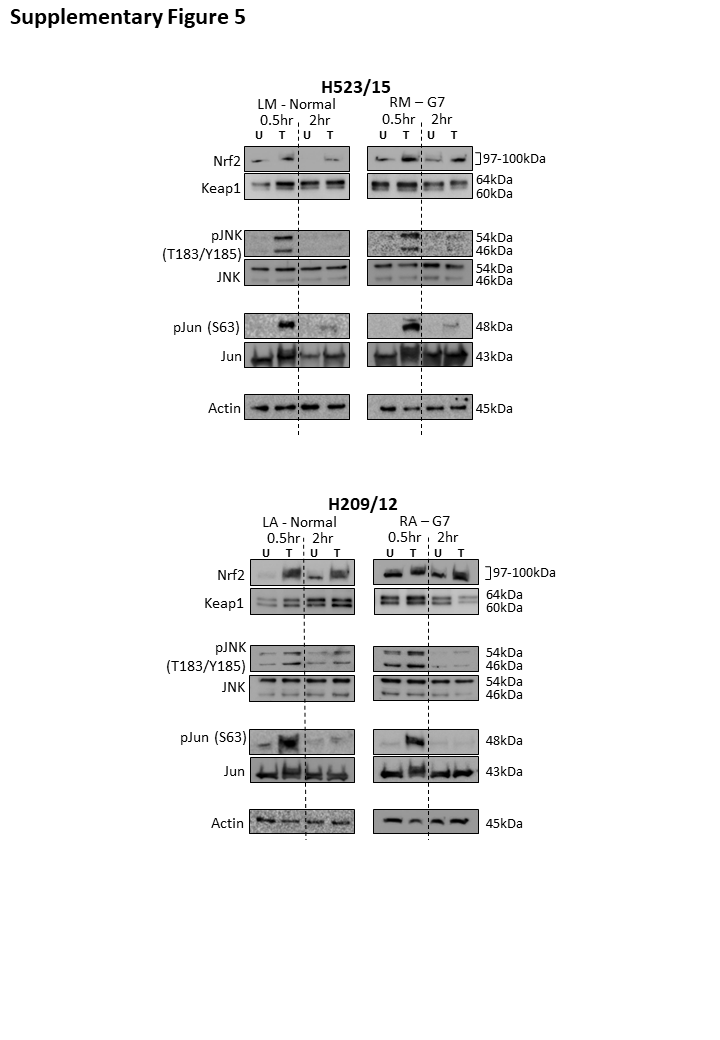

Supplement: Supplementary file 5 — Fig. S5. Low Temperature Plasma causes accumulation of Nrf2 and activation of AP‐1. [file FEB2-594-209-s005.tif]

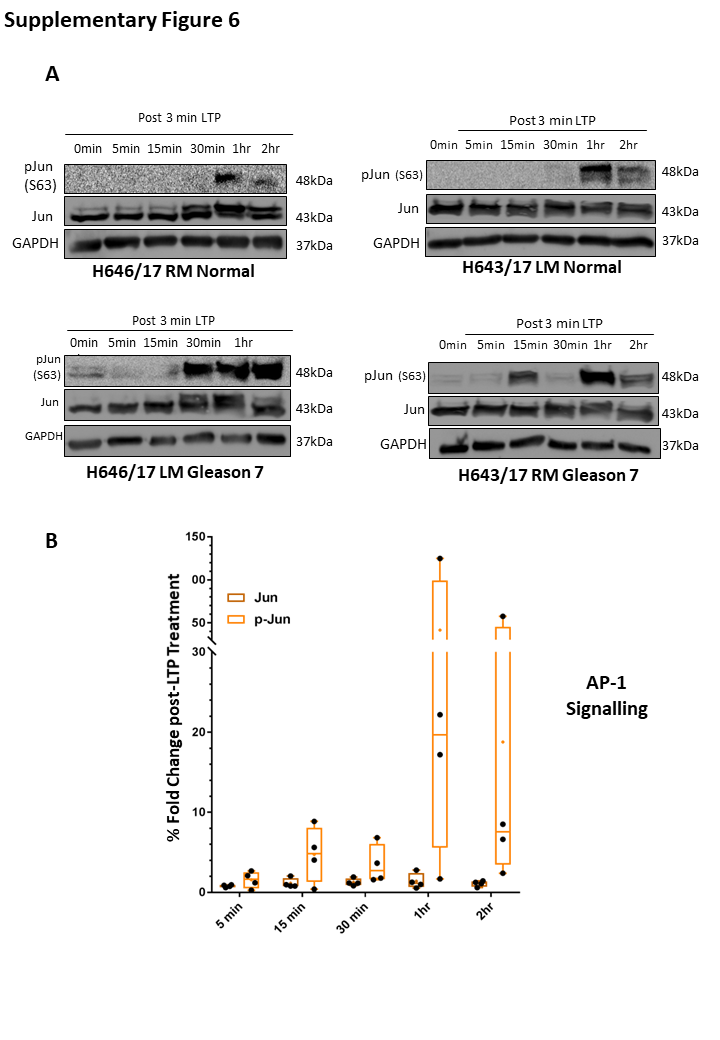

Supplement: Supplementary file 6 — Fig. S6. Low Temperature Plasma activates AP‐1 signalling in primary prostate epithelial cell cultures. [file FEB2-594-209-s006.tif]

Supplementary Figure 7

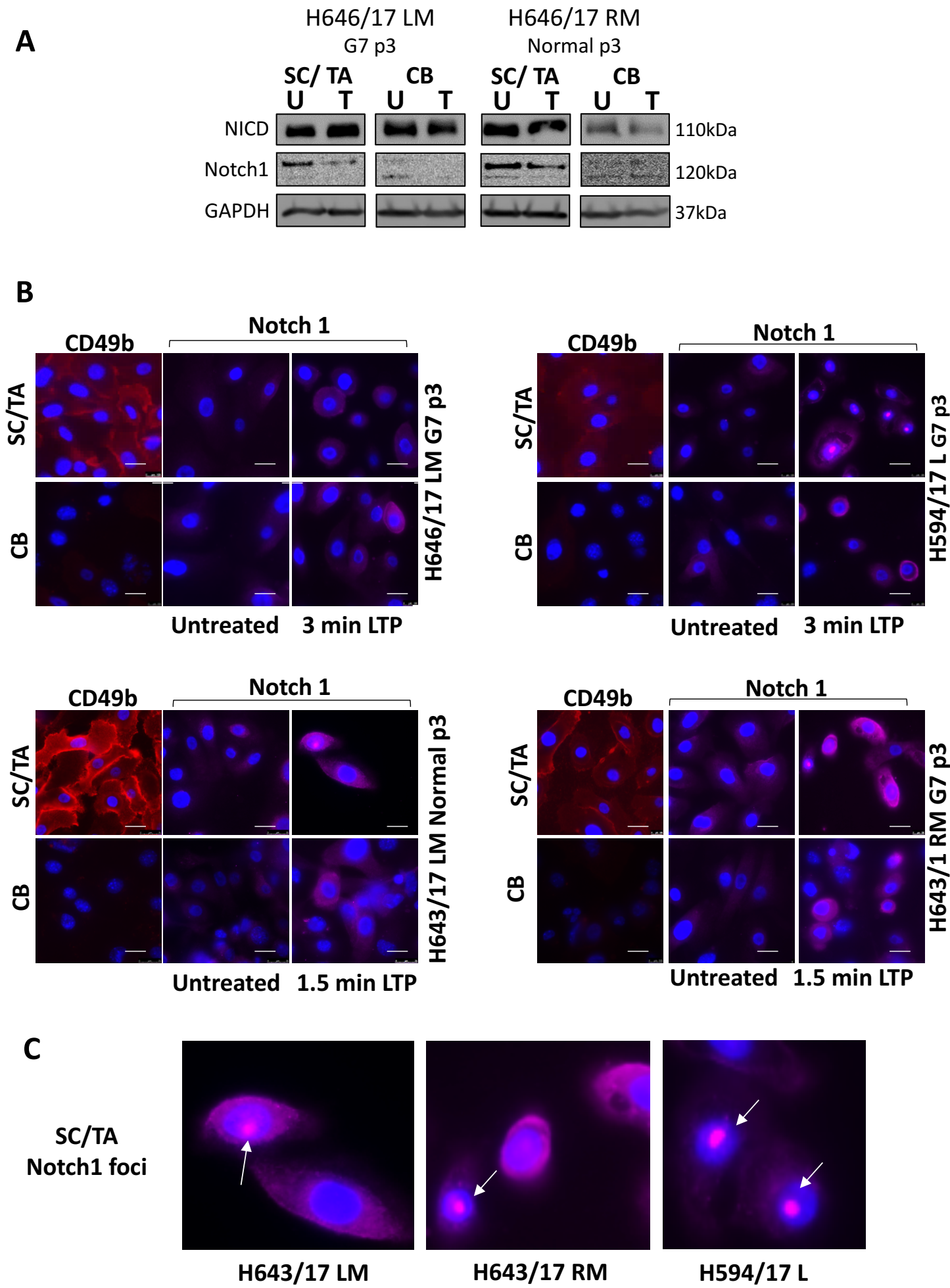

Supplement: Supplementary file 7 — Fig. S7. Notch signalling is more active in the SC/TA population, than in CB cells, after LTP treatment. [file FEB2-594-209-s007.pdf]

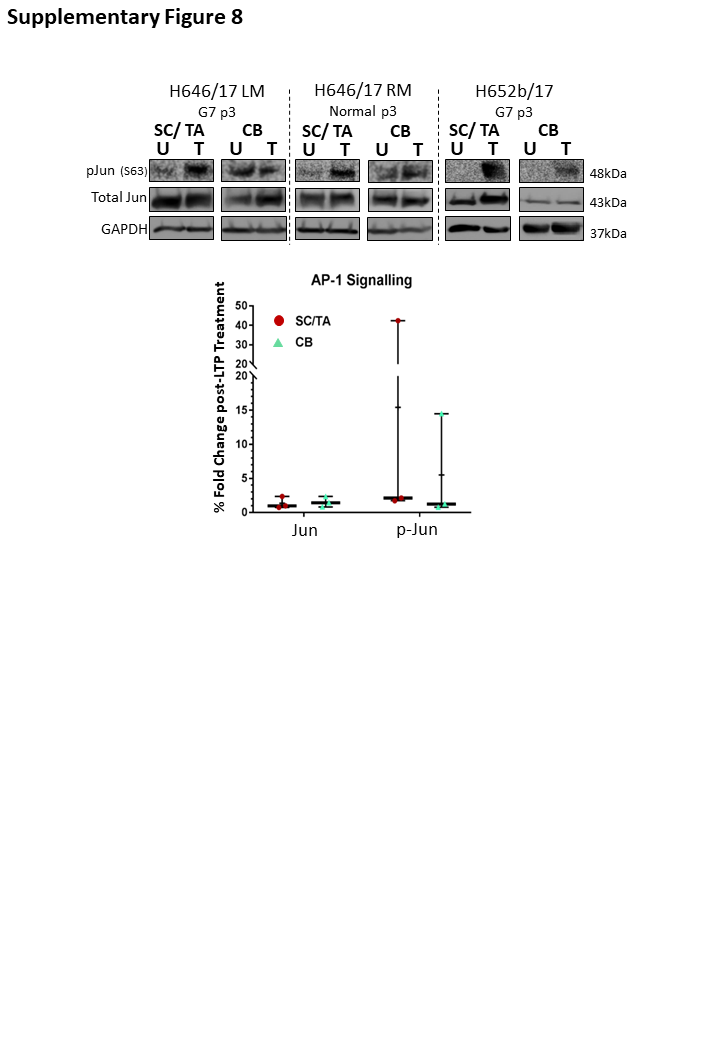

Supplement: Supplementary file 8 — Fig. S8. AP‐1 signalling is active in both the SC/TA cell population and the CB cell population after LTP treatment. [file FEB2-594-209-s008.tif]

Supplementary Figure 9

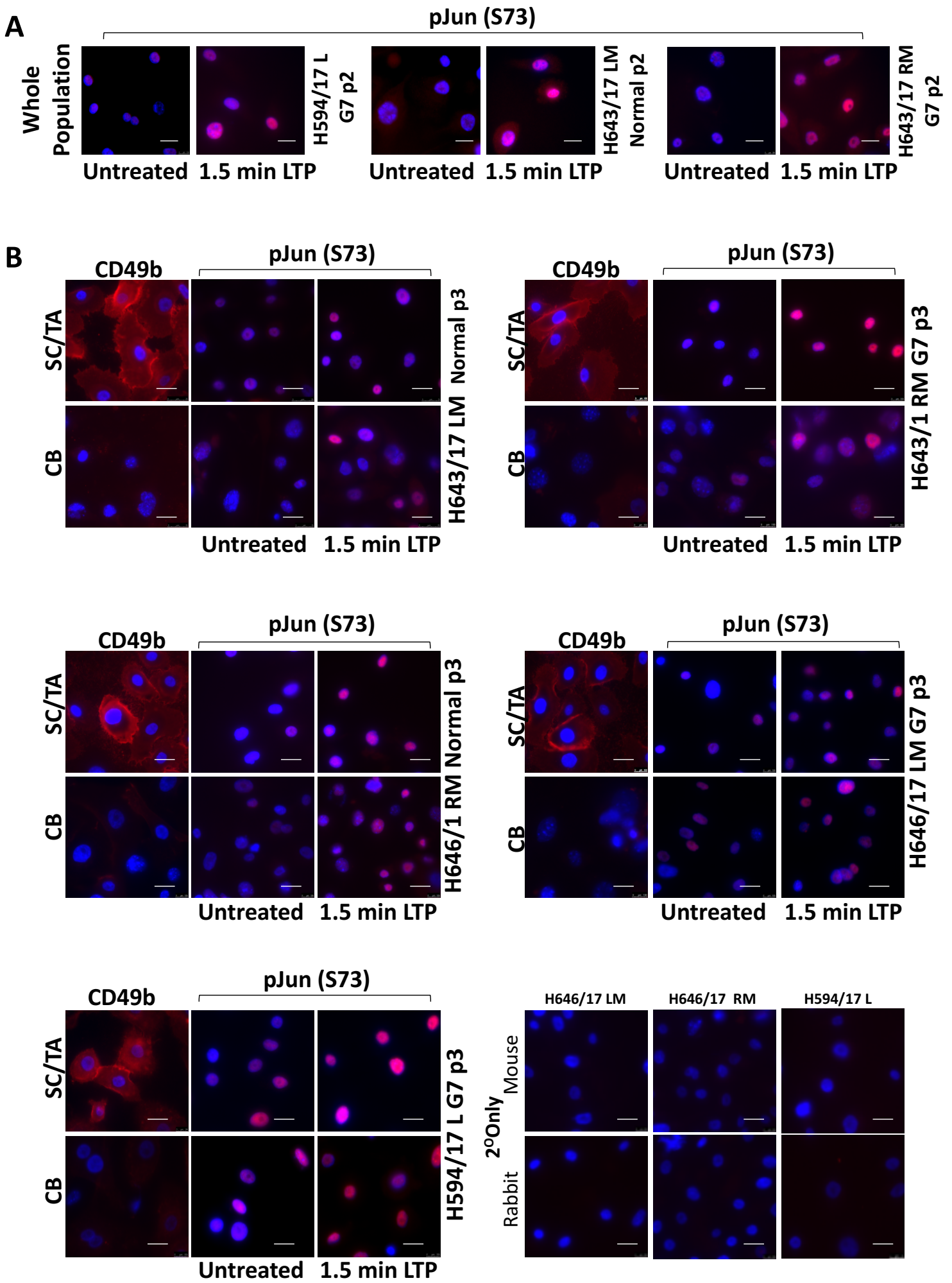

Supplement: Supplementary file 9 — Fig. S9. AP‐1 signalling is active in primary prostate epithelial cell cultures after LTP treatment. [file FEB2-594-209-s009.pdf]

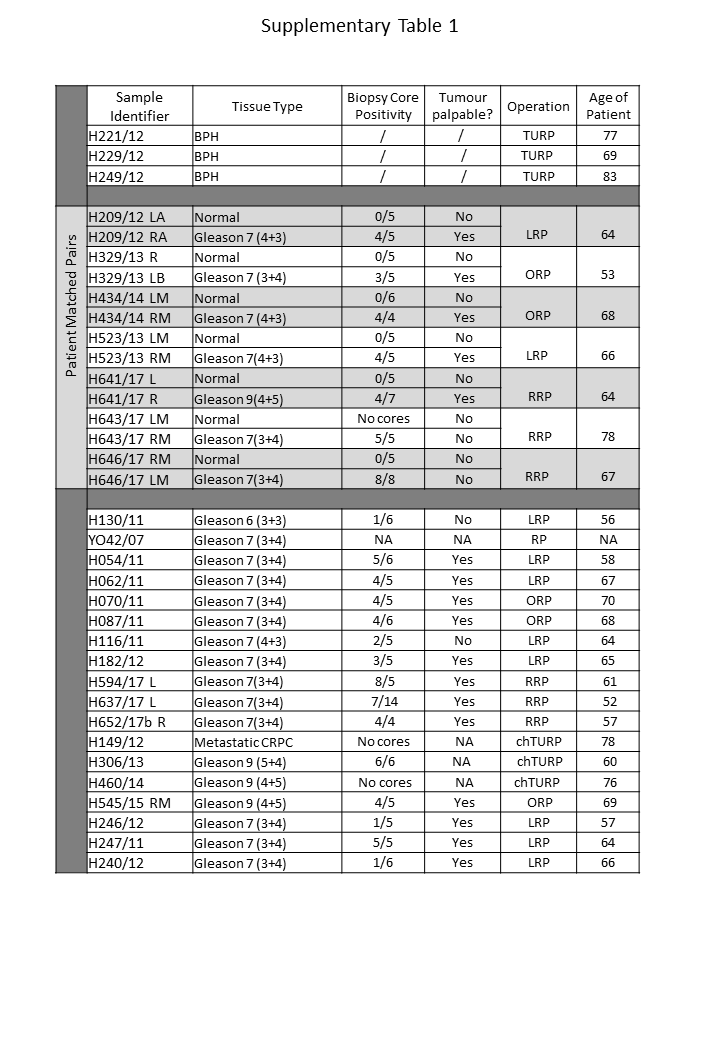

Supplement: Supplementary file 10 — Table S1. Patient information of all cell cultures used in the study. [file FEB2-594-209-s010.tif]

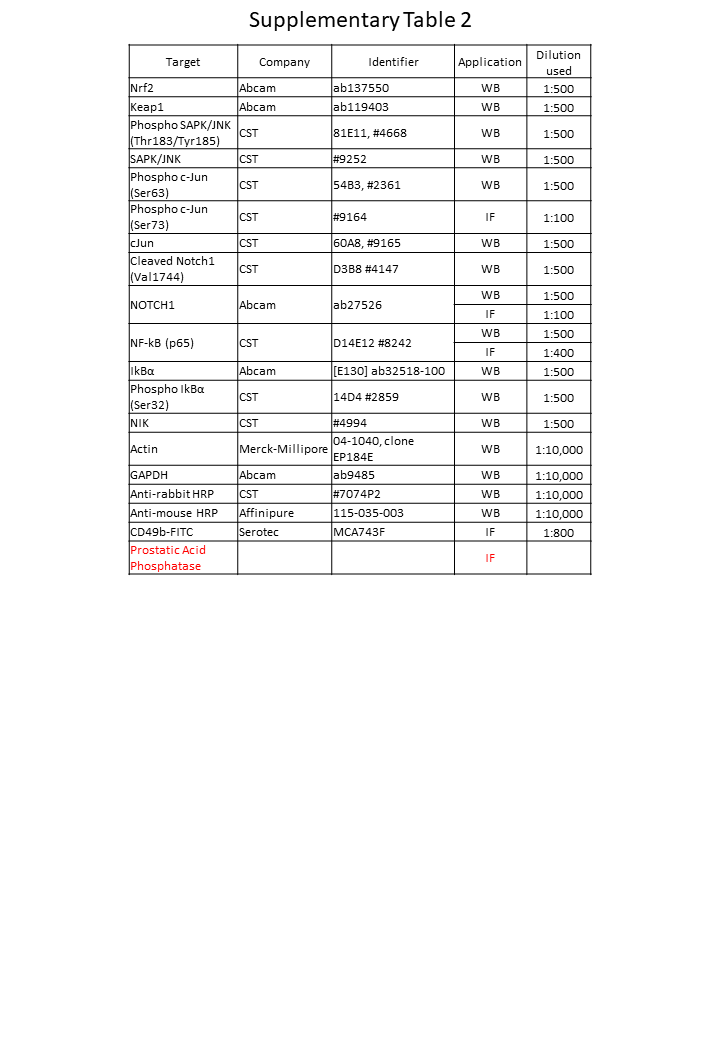

Supplement: Supplementary file 11 — Table S2. Antibodies used in the study. [file FEB2-594-209-s011.tif]

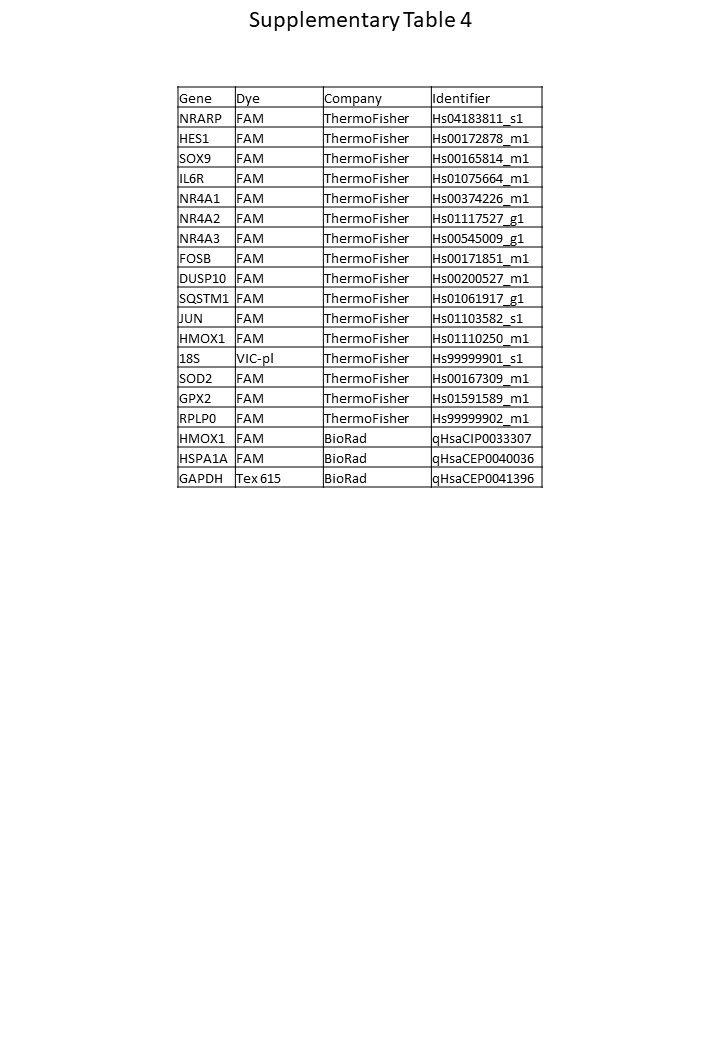

Supplement: Supplementary file 13 — Table S4. Taqman probes used in the study. [file FEB2-594-209-s013.tif]

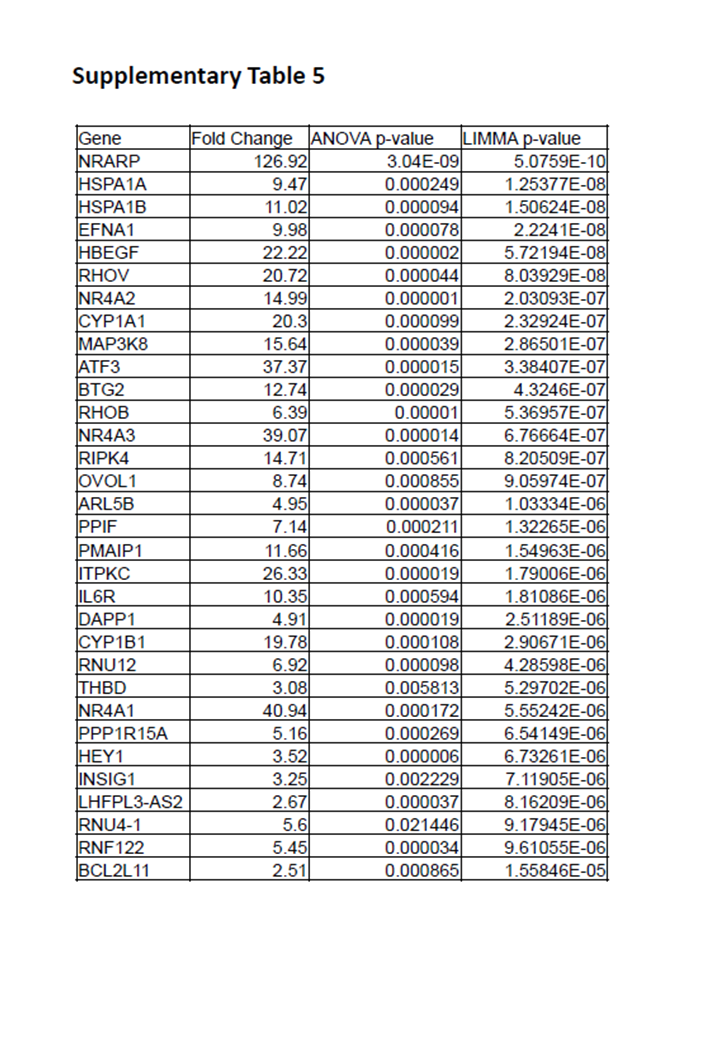

Supplement: Supplementary file 14 — Table S5. LIMMA meta‐analysis of ALL samples ‘treated against untreated'’ Excel file. [file FEB2-594-209-s014.tif]
