# Supplementary material for: Notch signalling is a potential resistance mechanism of progenitor cells within patient‐derived prostate cultures following ROS‐inducing treatments
Source: FEBS Lett. 2019 Sep 17;594(2):209–26. doi: 10.1002/1873-3468.13589 (PMC7003772; doi:10.1002/1873-3468.13589)
Supplement: Supplementary file 12 — Table S3. All samples ‘treated against untreated’ Excel file. [file FEB2-594-209-s012.pdf]

Transcript C	Treated Bi-	Untreated I	Fold Change (linear) (Treated vs. Untreated)	ANOVA p-v	FDR p-value	Gene Symbol	Description	Group	TC Comment
TC0900012	12.44	5.45	126.92	3.04E-09	0.000412	NRARP	NOTCH-regulated ankyrin repeat protein	Coding	
TC0100017	8.81	3.45	41.06	0.000002	0.050449			NonCoding	
TC1200007	12.61	7.25	40.94	0.000172	0.224222	NR4A1	nuclear receptor subfamily 4, group A, member 1 - Modulators of NFkB signalling	Multiple_Complex	
TC0900008	9.4	4.11	39.07	0.000014	0.079322	NR4A3	nuclear receptor subfamily 4, group A, member 3	Multiple_Complex	
TC0100011	13.2	7.98	37.37	0.000015	0.079322	ATF3	activating transcription factor 3 CREB family TF	Multiple_Complex	
TC1900008	11.99	7.09	29.86	0.000557	0.387514	FOSB	FBJ murine osteosarcoma viral oncogene homolog B - AP-1 Factor	Multiple_Complex	
TC1900008	11.64	6.92	26.33	0.000019	0.093426	ITPKC	inositol-trisphosphate 3-kinase C - involved in Ca2+ homeostasis	Multiple_Complex	
TC0700010	9.35	4.79	23.53	0.000071	0.14754	CTB-119C2.1	novel transcript antisense to NFE2L3 - 50% homology to Nrf2	NonCoding	
TC0100013	14.4	9.86	23.25	0.000096	0.165275	starawbo	Transcript Identified by AceView	Unassigned	
TC0500012	15.47	11	22.22	0.000002	0.050449	HBEGF	heparin-binding EGF-like growth factor - mitogen and cell chemotractant	Multiple_Complex	
TC1500009	11.04	6.67	20.72	0.000044	0.111767	RHOV	ras homolog family member V - modulates cytoskeleton by JNK activation	Multiple_Complex	
TC1500010	10.29	5.95	20.3	0.000099	0.165275	CYP1A1	cytochrome P450, family 1, subfamily A, polypeptide 1	Multiple_Complex	
TC0200016	9.9	5.59	19.78	0.000108	0.173118	CYP1B1	cytochrome P450, family 1, subfamily B, polypeptide 1 - removes cellular oxygenation products and decreases oxidative stress	Multiple_Complex	
TC1700012	11.99	7.78	18.43	0.004045	0.775973	ZNF750	zinc finger protein 750 p63 target gene	Coding	
TC0100017	14.46	10.3	17.82	0.000111	0.173263			NonCoding	
TC1700010	8.68	4.56	17.38	0.000215	0.247197	titari	Transcript Identified by AceView	Coding	
TC0200008	10.13	6.02	17.25	0.002385	0.669795	IL36G	interleukin 36, gamma - aids local immune response through MAPK and NF-KB activation	Coding	
TC0600007	8.8	4.69	17.25	0.000902	0.47326	ZSCAN12P1	zinc finger and SCAN domain containing 12 pseudogene 1	Multiple_Complex	
TC0100007	14.08	9.97	17.24	0.000135	0.197417	flawrobu	Transcript Identified by AceView	Unassigned	
TC0200010	14.7	10.63	16.76	0.004065	0.775973	CCL20	chemokine (C-C motif) ligand 20 - chemotractant for lymphocytes and neutrophils	Multiple_Complex	
TC1000007	11.04	7.07	15.64	0.000039	0.104367	MAP3K8	mitogen-activated protein kinase kinase kinase 8 - activates MAPK and NF-KB	Multiple_Complex	
TC0200014	8.4	4.49	14.99	0.000001	0.050449	NR4A2	nuclear receptor subfamily 4, group A, member 2	Multiple_Complex	
TC2100008	12.51	8.63	14.71	0.000561	0.38867	RIPK4	receptor-interacting serine-threonine kinase 4 - p63 target gene activates NF-kB	Coding	
TC0100007	12.87	9.11	13.54	0.000081	0.150832	beepabu	Transcript Identified by AceView	Unassigned	
TC0600007	8.77	5.01	13.53	0.000853	0.463556	CD83	CD83 molecule - involved in antigen presentation	Multiple_Complex	
TC1800007	7.37	3.69	12.83	0.000405	0.325191	tawry	Transcript Identified by AceView	Unassigned	
TC0100011	13.75	10.08	12.74	0.000029	0.097189	BTG2	BTG family, member 2 -ve inhibtor of the cell cycle	Multiple_Complex	
TC1100009	8.91	5.26	12.57	2.75E-08	0.001863	spychey	Transcript Identified by AceView	Coding	
TC1500010	12.93	9.3	12.4	0.015026	0.99496	SEMA7A	semaphorin 7A, GPI membrane anchor (John Milton Hagen blood group)	Multiple_Complex	
TC0800009	8.51	4.91	12.14	0.005419	0.836397	EGR3	early growth response 3	Multiple_Complex	
TC1800007	10.23	6.69	11.66	0.000416	0.32985	PMAIP1	phorbol-12-myristate-13-acetate-induced protein 1 - NOXA	Multiple_Complex	
TC1600009	13.35	9.84	11.37	0.007988	0.907048	PRSS22	protease, serine, 22	Multiple_Complex	
TC0800011	9.25	5.77	11.18	0.000021	0.097189	GEM	GTP binding protein overexpressed in skeletal muscle	Multiple_Complex	
TC0600007	12.36	8.9	11.02	0.000094	0.165275	HSPA1B	heat shock 70kDa protein 1B; heat shock 70kDa protein 1A	Coding	
TC0100011	16.1	12.73	10.37	0.000047	0.113669			NonCoding	
TC0100010	9.75	6.38	10.35	0.000594	0.396695	IL6R	interleukin 6 receptor - NF-kB	Multiple_Complex	
TC0100010	10.82	7.5	9.98	0.000078	0.150832	EFNA1	ephrin-A1	Multiple_Complex	
TC0100015	9.35	6.05	9.88	0.004145	0.776625			NonCoding	
TC0600007	12.7	9.45	9.47	0.000249	0.267205	HSPA1A	heat shock 70kDa protein 1A; heat shock 70kDa protein 1B inhibits MAPK activation	Coding	
TC0600007	8.77	5.62	8.88	0.000587	0.394786	ZNF165	zinc finger protein 165	Coding	
TC1100008	9.04	5.91	8.74	0.000855	0.463556	OVOL1	ovo-like zinc finger 1	Multiple_Complex	
TC0900006	6.92	3.94	7.91	0.002	0.631322	bersta	Transcript Identified by AceView	Unassigned	
TC0100017	11.72	8.76	7.75	0.000669	0.412704	DUSP10	dual specificity phosphatase 10	Multiple_Complex	
TC1700006	12.95	10.07	7.36	0.003929	0.772902	KDM6B	lysine (K)-specific demethylase 6B - H3K27me3	Multiple_Complex	
TC0100015	8.27	5.42	7.2	0.000073	0.149418			NonCoding	
TC1000008	14.29	11.45	7.14	0.000211	0.245464	PPIF	peptidylprolyl isomerase F	Multiple_Complex	
TC0600014	8.61	5.78	7.12	0.021589	0.999955	SERPINB9	serpin peptidase inhibitor, clade B (ovalbumin), member 9	Multiple_Complex	
TC1700007	10.79	7.97	7.04	0.000107	0.173118			NonCoding	
TC1400008	5.63	2.82	7.02	0.001131	0.525631			NonCoding	
TC0100017	13.35	10.55	6.94	0.002932	0.717301	C1orf74	chromosome 1 open reading frame 74	Coding	
TC2200007	11.23	8.44	6.92	0.000098	0.165275	RNU12	RNA, U12 small nuclear	Multiple_Complex	
TC0100007	8.96	6.21	6.74	0.000109	0.173118	fluweybo	Transcript Identified by AceView	Coding	
TC0600011	6.39	3.63	6.74	0.000096	0.165275	LOC100287365	Transcript Identified by AceView, Entrez Gene ID(s) 100287365	Coding	
TC0300013	8.03	5.29	6.69	0.010059	0.94893	slukeebu	Transcript Identified by AceView	Coding	
TC0200006	13.51	10.83	6.39	0.00001	0.069027	RHOB	ras homolog family member B - can mediate apoptosis after DNA damage	Coding	
TC0600008	15.93	13.36	5.96	0.000486	0.352536	PNRC1	proline-rich nuclear receptor coactivator 1	Multiple_Complex	
TC0100007	13.76	11.21	5.85	0.005315	0.834256	ZC3H12A	zinc finger CCCH-type containing 12A; microRNA 6732	Multiple_Complex	
TC0100013	14.06	11.52	5.8	0.00132	0.56359	ID3	inhibitor of DNA binding 3, dominant negative helix-loop-helix protein	Multiple_Complex	
TC0300008	5.94	3.42	5.77	0.00006	0.13038	neebubu	Transcript Identified by AceView	Unassigned	
TC1900010	14.46	11.94	5.72	0.000117	0.178757	PLAUR	plasminogen activator, urokinase receptor	Multiple_Complex	
TC0600009	11	8.5	5.68	0.000408	0.325854	NCOA7	nuclear receptor coactivator 7	Multiple_Complex	
TC0100014	13.99	11.5	5.62	0.000081	0.150832	JUN	jun proto-oncogene	Multiple_Complex	
TC1200012	14.32	11.83	5.6	0.021446	0.999955	RNU4-1	RNA, U4 small nuclear 1	Small_RNA	
TC0800010	9.88	7.43	5.45	0.000034	0.100368	RNF122	ring finger protein 122	Multiple_Complex	
TC1700007	5.43	2.98	5.45	0.003229	0.744794			NonCoding	
TC0600007	10.23	7.79	5.42	0.002235	0.65526	mogluby	Transcript Identified by AceView	Coding	
TC0X00009	10.27	7.84	5.4	0.000862	0.463556	KLHL15	kelch-like family member 15	Coding	
TC1900008	11.1	8.74	5.16	0.000269	0.274512	PPP1R15A	protein phosphatase 1, regulatory subunit 15A	Multiple_Complex	
TC0600011	9.89	7.54	5.13	0.001018	0.501693			NonCoding	
TC0700010	8.51	6.16	5.12	0.000222	0.249275			NonCoding	
TC2200009	8.06	5.71	5.08	0.003075	0.734859	APOBEC3B	apolipoprotein B mRNA editing enzyme, catalytic polypeptide-like 3B	Coding	
TC0100017	8	5.66	5.07	0.0006	0.397331	LOC101930114	uncharacterized LOC101930114; novel transcript	NonCoding	
TC1000007	5.88	3.56	4.98	0.046732	0.999955	peynyby	Transcript Identified by AceView	Coding	
TC1000006	14.07	11.77	4.95	0.000037	0.103191	ARL5B	ADP-ribosylation factor like GTPase 5B	Multiple_Complex	
TC0400008	11.29	9	4.91	0.000019	0.093426	DAPP1	dual adaptor of phosphotyrosine and 3-phosphoinositides	Multiple_Complex	
TC1300008	14.61	12.32	4.91	0.00001	0.069027	TSC22D1	TSC22 domain family, member 1	Multiple_Complex	

TC1100009	5.5	3.21	4.89	0.00001	0.069027	shakloy	Transcript Identified by AceView	Coding
TC1400008	15.31	13.03	4.84	0.001085	0.520174	NFKBIA	nuclear factor of kappa light polypeptide gene enhancer in B-cells inhibitor, alpha	Multiple_Complex
TC1200011	12.86	10.59	4.82	0.001178	0.538542	TMCC3	transmembrane and coiled-coil domain family 3; microRNA 7844	Multiple_Complex
TC1100009	6.51	4.25	4.8	0.000441	0.334608	temi	Transcript Identified by AceView	Unassigned
TC1100011	14.67	12.41	4.8	0.002221	0.65526	FOSL1	FOS-like antigen 1	Multiple_Complex
TC0500010	6.93	4.7	4.7	0.001808	0.608799	Y_RNA	Y RNA [Source:RFAM;Acc:RF00019]	NonCoding
TC0300008	7.43	5.2	4.69	0.000054	0.122153	RP11-757F18.5	novel transcript, antisense to GCET2	NonCoding
TC1700008	15.17	12.95	4.67	0.000321	0.295523	SOX9	SRY box 9	Multiple_Complex
TC1100008	10.2	7.98	4.66	0.015033	0.99496	ZC3H12C	zinc finger CCCH-type containing 12C	Multiple_Complex
TC0700008	10.51	8.29	4.64	0.000755	0.443965	shorpaby	Transcript Identified by AceView	Unassigned
TC0500012	9.23	7.04	4.59	0.00405	0.775973	IRF1	interferon regulatory factor 1	Multiple_Complex
TC2000008	9.2	7	4.59	0.006748	0.880199	SNORD17	small nucleolar RNA, C/D box 17	Small_RNA
TC1700010	9.21	7.02	4.55	0.02647	0.999955	CTD-3014M21.1	TEC	NonCoding
TC1800007	5.83	3.65	4.54	0.012348	0.96061	neeser	Transcript Identified by AceView	Unassigned
TC1700008	6.37	4.19	4.53	0.031339	0.999955	loysa	Transcript Identified by AceView	Unassigned
TC0600011	13.93	11.76	4.49	0.000613	0.404263	PPP1R10	protein phosphatase 1, regulatory subunit 10	Multiple_Complex
TC0600011	14.23	12.08	4.43	0.000043	0.110077	RNU1-88P	RNA, U1 small nuclear 88, pseudogene [Source:HGNC Symbol;Acc:HGNC:48430]	Small_RNA
TC1000010	9.16	7.02	4.4	0.000584	0.394441	joymorby	Transcript Identified by AceView	Unassigned
TC1900007	9.4	7.27	4.38	0.005788	0.848027	PLEKHF1	pleckstrin homology domain containing, family F (with FYVE domain) member 1	Coding
TC0100012	10.16	8.04	4.35	0.000192	0.239224	KLHL21	kelch-like family member 21	Multiple_Complex
TC0900009	8.16	6.04	4.34	0.003824	0.770736	LURAP1L-AS1	LURAP1L antisense RNA 1	NonCoding
TC1200012	12.55	10.44	4.34	0.008134	0.909535	RNU4-2	RNA, U4 small nuclear 2	Multiple_Complex
TC1000009	8.08	5.97	4.32	0.038252	0.999955			NonCoding
TC1600010	8.8	6.69	4.32	0.018785	0.999955	RRAD	Ras-related associated with diabetes	Multiple_Complex
TC0100015	14.4	12.33	4.2	0.000027	0.097189	RNU1-13P	RNA, U1 small nuclear 13, pseudogene	Small_RNA
TC0100015	17.19	15.14	4.14	0.000011	0.069027	U1	U1 spliceosomal RNA [Source:RFAM;Acc:RF00003]	Small_RNA
TC0600011	6.14	4.09	4.14	0.010406	0.950398			NonCoding
TC1100011	11.68	9.64	4.11	0.000077	0.150832	NXF1	nuclear RNA export factor 1	Multiple_Complex
TC0300006	9.2	7.17	4.1	0.027495	0.999955	IRAK2	interleukin 1 receptor associated kinase 2	Multiple_Complex
TC0700007	14.2	12.17	4.1	0.015204	0.995547	CLDN4	claudin 4	Multiple_Complex
TC0X00010	11.02	8.99	4.1	0.000639	0.404488	GLA	galactosidase, alpha	Multiple_Complex
TC0200007	7.5	5.47	4.08	0.00553	0.836397	RP11-373D23.3	novel transcript	NonCoding
TC0500007	8.15	6.13	4.08	0.000183	0.233903			NonCoding
TC1900010	9.3	7.28	4.07	0.012237	0.96061	JUND	jun D proto-oncogene	Coding
TC0300009	6.49	4.48	4.02	0.035602	0.999955			NonCoding
TC0600007	5.86	3.86	4.02	0.006301	0.868734	beykleebu	Transcript Identified by AceView	Coding
TC0200006	8.35	8.35	4	0.044476	0.999955	ID2	inhibitor of DNA binding 2, dominant negative helix-loop-helix protein	Multiple_Complex
TC0800011	7.82	5.83	3.98	0.002669	0.701278			NonCoding
TC0100009	12.39	10.4	3.96	0.006495	0.870775	U1	U1 spliceosomal RNA [Source:RFAM;Acc:RF00003]	Small_RNA
TC0300012	14.29	12.31	3.95	0.000022	0.097189	CCNL1	cyclin L1	Multiple_Complex
TC0600009	11.65	9.69	3.9	0.000197	0.240745	RNU1-18P	RNA, U1 small nuclear 18, pseudogene [Source:HGNC Symbol;Acc:HGNC:41944]	Small_RNA
TC1100011	5.98	4.02	3.9	0.009343	0.930282	PYGM	phosphorylase, glycogen, muscle	Multiple_Complex
TC0600009	17.43	15.47	3.89	0.000026	0.097189	TNFAIP3	tumor necrosis factor, alpha-induced protein 3	Multiple_Complex
TC0600013	9.33	7.39	3.83	0.000327	0.295523			NonCoding
TC0100015	12.97	11.05	3.78	0.000026	0.097189	RNVU1-17	RNA, variant U1 small nuclear 17	Small_RNA
TC2100007	15.65	13.75	3.73	0.008322	0.913881	ETS2	v-ets avian erythroblastosis virus E26 oncogene homolog 2	Multiple_Complex
TC0500011	7.6	5.7	3.72	0.003988	0.775973	LOC647859	Transcript Identified by AceView, Entrez Gene ID(s) 647859	Unassigned
TC0600011	7.84	5.95	3.72	0.000966	0.485943	RP1-30M3.5	novel transcript antisense to ACOT13	NonCoding
TC1800007	15.97	14.07	3.72	0.014129	0.983937	NEDD4L	neural precursor cell expressed, developmentally down-regulated 4-like, E3 ubiquitin protein ligase	Multiple_Complex
TC0400008	11.54	9.65	3.71	0.000821	0.463556	FOSL1P1	FOS-like antigen 1 pseudogene 1 [Source:HGNC Symbol;Acc:HGNC:44055]	Pseudogene
TC0100016	7.26	5.37	3.7	0.0079	0.902202			NonCoding
TC1000007	10.53	8.67	3.64	0.005683	0.84631	SIRT1	sirtuin 1	Multiple_Complex
TC2000008	6.93	5.07	3.62	0.00202	0.633479	loytar	Transcript Identified by AceView	Unassigned
TC1900008	5.96	4.11	3.61	0.000638	0.404488			NonCoding
TC1700010	16.66	14.82	3.59	0.000003	0.061095	SNORD3D	small nucleolar RNA, C/D box 3D	Multiple_Complex
TC0100018	12.17	10.33	3.58	0.00946	0.93154	DENND2C	DENN/MADD domain containing 2C	Multiple_Complex
TC0100008	9.66	7.82	3.57	0.005327	0.834256	PDE4B	phosphodiesterase 4B, cAMP-specific	Multiple_Complex
TC0100008	5.78	3.95	3.54	0.011364	0.959571	gorzawbo	Transcript Identified by AceView	Coding
TC0300008	5.15	3.33	3.54	0.005364	0.834469			NonCoding
TC0900010	6.24	4.42	3.54	0.000932	0.481797			NonCoding
TC1400010	5.37	3.54	3.54	0.001077	0.520174	RN7SL472P	RNA, 7SL, cytoplasmic 472, pseudogene [Source:HGNC Symbol;Acc:HGNC:46488]	NonCoding
TC1000006	8.39	6.57	3.53	0.001965	0.627868	PFKFB3	6-phosphofructo-2-kinase/fructose-2,6-biphosphatase 3	Multiple_Complex
TC0100016	6.16	4.35	3.52	0.03463	0.999955			NonCoding
TC0800010	6.68	4.87	3.52	0.000006	0.068513	HEY1	hes-related family bHLH transcription factor with YRPW motif 1	Multiple_Complex
TC1200010	8.05	6.24	3.52	0.004018	0.775973			NonCoding
TC0800007	6.45	4.64	3.5	0.006067	0.862705	smakor	Transcript Identified by AceView	NonCoding
TC0800011	7.85	6.05	3.5	0.016745	0.999955			NonCoding
TC1400006	6.54	4.74	3.49	0.005545	0.836397	toyslu	Transcript Identified by AceView	Coding
TC1100013	9.36	7.56	3.48	0.004332	0.781669	CLCF1	cardiotrophin-like cytokine factor 1	Coding
TC0900010	12.58	10.8	3.46	0.011331	0.959571	NFIL3	nuclear factor, interleukin 3 regulated	Coding
TC1000011	7.38	5.58	3.46	0.001565	0.583987	C10orf55	chromosome 10 open reading frame 55	Multiple_Complex
TC0100011	4.68	2.9	3.44	0.002224	0.65526			NonCoding
TC0600013	5.89	4.11	3.43	0.018985	0.999955			NonCoding
TC1200011	12.39	10.61	3.43	0.000149	0.21297	RNU1-83P	RNA, U1 small nuclear 83, pseudogene [Source:HGNC Symbol;Acc:HGNC:48425]	Small_RNA
TC0400008	5.18	3.4	3.42	0.0002	0.240745			NonCoding
TC0700011	5.24	3.47	3.42	0.000097	0.165275	weesleybu	Transcript Identified by AceView	Unassigned
TC1900009	16.59	14.83	3.4	0.00001	0.069027	DNAJB1	DnaJ (Hsp40) homolog, subfamily B, member 1	Multiple_Complex

TC0100013	12.83	11.07	3.38	0.002245	0.656116	SNORA44	small nucleolar RNA, H/ACA box 44; small nucleolar RNA, H/ACA box 61; small nucleolar RNA, H/ACA box 16A; small nucleolar RNA host gene 12	Multiple_Complex
TC1500008	9.12	7.37	3.38	0.022684	0.999955	RNU6-132P	RNA, U6 small nuclear 132, pseudogene [Source:HGNC Symbol;Acc:HGNC:47095]	Small_RNA
TC0100015	5.93	4.18	3.37	0.006414	0.868734	RNU6-1318P	RNA, U6 small nuclear 1318, pseudogene [Source:HGNC Symbol;Acc:HGNC:48281]	Small_RNA
TC1800007	7.6	5.84	3.37	0.000372	0.310175	RP11-845C23.2	novel transcript, sense intronic to NEDD4L	NonCoding
TC0100007	18.28	16.53	3.36	0.000007	0.068513	RNU1-4	RNA, U1 small nuclear 4	Small_RNA
TC0100007	18.28	16.53	3.36	0.000007	0.068513	RNU1-2	RNA, U1 small nuclear 2	Small_RNA
TC0100009	18.28	16.53	3.36	0.000007	0.068513	RNVU1-18	Homo sapiens RNA, variant U1 small nuclear 18 (RNVU1-18), small nuclear RNA.; Homo sapiens RNA, U1 small nuclear 28, pseudogene (RNU1-28P), small nuclea	Small_RNA
TC1000011	8.79	7.04	3.36	0.002783	0.70472	ITPRIP	inositol 1,4,5-trisphosphate receptor interacting protein	Multiple_Complex
TC1400006	18.28	16.53	3.36	0.000007	0.068513	RNU1-27P	RNA, U1 small nuclear 27, pseudogene	Small_RNA
TC0600013	13.68	11.94	3.33	0.00398	0.775973	SOD2	superoxide dismutase 2, mitochondrial	Multiple_Complex
TC1700007	4.72	2.99	3.33	0.02668	0.999955			NonCoding
TC0200015	9.71	7.97	3.32	0.000655	0.407614	DNAJB1P1	DnaJ heat shock protein family (Hsp40) member B1 pseudogene 1 [Source:HGNC Symbol;Acc:HGNC:24988]	Pseudogene
TC0200009	13.62	11.89	3.3	0.000061	0.131328	RNU4ATAC	RNA, U4atac small nuclear (U12-dependent splicing)	Multiple_Complex
TC0100015	16.52	14.8	3.28	0.000161	0.217793	U1	U1 spliceosomal RNA [Source:RFAM;Acc:RF00003]	Small_RNA
TC0400011	17.44	15.73	3.28	0.000079	0.150832	RNU1-89P	RNA, U1 small nuclear 89, pseudogene [Source:HGNC Symbol;Acc:HGNC:48431]	Small_RNA
TC0300009	7.18	5.47	3.27	0.003715	0.763778	LOC344887	NmrA-like family domain containing 1 pseudogene	Multiple_Complex
TC1400008	6.66	4.95	3.27	0.003758	0.763778			NonCoding
TC0100018	12.94	11.25	3.25	0.000258	0.271975	IER5	immediate early response 5	Coding
TC0700009	12.89	11.19	3.25	0.002229	0.65526	INSIG1	insulin induced gene 1	Multiple_Complex
TC0800008	5.69	3.98	3.25	0.00299	0.724087	RP11-79H23.3	novel transcript	NonCoding
TC0900006	15.14	13.45	3.24	0.000002	0.050449	DNAJA1	DnaJ (Hsp40) homolog, subfamily A, member 1	Multiple_Complex
TC0700013	13.56	11.87	3.23	0.012294	0.96061	IFRD1	interferon-related developmental regulator 1	Multiple_Complex
TC0200016	8.09	6.41	3.21	0.034702	0.999955	PER2	period circadian clock 2	Multiple_Complex
TC1700011	9.48	7.8	3.21	0.0468	0.999955	U3	Small nucleolar RNA U3 [Source:RFAM;Acc:RF00012]	Multiple_Complex
TC0900011	7.37	5.69	3.2	0.002651	0.698798	KLF4	Kruppel-like factor 4 (gut)	Multiple_Complex
TC0400008	7.17	5.51	3.16	0.018449	0.999955			NonCoding
TC0900009	10.68	9.02	3.16	0.000054	0.122153			NonCoding
TC0100015	6.51	4.85	3.15	0.008462	0.915165	U4	U4 spliceosomal RNA [Source:RFAM;Acc:RF00015]	Small_RNA
TC1100008	13	11.34	3.15	0.000123	0.184123	PCF11	PCF11 cleavage and polyadenylation factor subunit	Multiple_Complex
TC0900008	8.97	7.32	3.14	0.002748	0.701278	ZBTB43	zinc finger and BTB domain containing 43	Multiple_Complex
TC2000006	5.46	3.81	3.14	0.004684	0.789954			NonCoding
TC0500009	14.77	13.12	3.13	0.000354	0.304943	SQSTM1	sequestosome 1	Multiple_Complex
TC0900010	8.18	6.53	3.13	0.043938	0.999955	KLF9	Kruppel-like factor 9	Multiple_Complex
TC0500013	12.12	10.48	3.12	0.000165	0.219139	FAM53C	family with sequence similarity 53, member C	Multiple_Complex
TC1900009	4.75	3.12	3.11	0.015285	0.998513	woypu	Transcript Identified by AceView	Unassigned
TC0500007	13.46	11.82	3.1	0.01281	0.963788	OCLN	occludin	Multiple_Complex
TC0600011	5.91	4.27	3.1	0.001349	0.565911			NonCoding
TC1900006	9.64	8.02	3.08	0.0395	0.999955	GADD45B	growth arrest and DNA-damage-inducible, beta	Multiple_Complex
TC2000008	4.97	3.35	3.08	0.005813	0.848457	THBD	thrombomodulin	Coding
TC1100012	8.94	7.32	3.07	0.00198	0.628151	SNORD14E	small nucleolar RNA, C/D box 14E	Small_RNA
TC1500006	11.23	9.61	3.07	0.003332	0.744794	VPS18	VPS18 CORVET/HOPS core subunit	Multiple_Complex
TC2000008	6.29	4.67	3.06	0.014345	0.983937	SNX5	Zhang2013 ALT_ACCEPTOR, ALT_DONOR, coding, INTERNAL, intronic best transcript NM_152227	NonCoding
TC0500007	12.65	11.04	3.05	0.000009	0.069027	F2RL1	coagulation factor II (thrombin) receptor-like 1	Multiple_Complex
TC0600009	8.12	6.51	3.05	0.033136	0.999955	SNORA33	small nucleolar RNA, H/ACA box 33	Small_RNA
TC0600011	7.73	6.12	3.05	0.001741	0.606851			NonCoding
TC1000008	8.26	6.65	3.05	0.014505	0.989069			NonCoding
TC2000007	9.48	7.87	3.05	0.000031	0.097189	CEBPB	CCAAT/enhancer binding protein (C/EBP), beta	Multiple_Complex
TC0100015	12.45	10.85	3.04	0.000009	0.069027	RNVU1-1	RNA, variant U1 small nuclear 1	Small_RNA
TC0200016	7.43	5.83	3.03	0.001231	0.5499	GPR75	G protein-coupled receptor 75	Coding
TC0800009	4.38	2.78	3.02	0.001895	0.616866	CTD-3247F14.2	Transcript Identified by AceView; novel transcript, overlapping to EGR3	Multiple_Complex
TC0800011	14.01	12.42	3.01	0.005681	0.84631	KLF10	Kruppel-like factor 10	Multiple_Complex
TC0100006	11.46	9.87	3	0.000525	0.371143	SPSB1	splA/ryanodine receptor domain and SOCS box containing 1	Multiple_Complex
TC0900008	6.66	5.07	3	0.004343	0.781669	SLC25A25	solute carrier family 25 (mitochondrial carrier; phosphate carrier), member 25	Multiple_Complex
TC1300008	5.54	3.95	3	0.0354	0.999955			NonCoding
TC1700012	10.88	9.29	3	0.003328	0.744794	LINC00673	long intergenic non-protein coding RNA 673; long intergenic non-protein coding RNA 511	Multiple_Complex
TC0300009	16.57	14.99	2.99	0.00924	0.928803	HES1	hes family bHLH transcription factor 1	Multiple_Complex
TC1800007	14.05	12.48	2.98	0.004049	0.775973	LIPG	lipase, endothelial	Multiple_Complex
TC0700007	5.26	3.69	2.97	0.000379	0.310273	RP11-339F13.2		Multiple_Complex
TC0100015	18.29	16.72	2.96	0.000026	0.097189	RNVU1-7	RNA, variant U1 small nuclear 7	Small_RNA
TC0X00007	11.37	9.8	2.96	0.016805	0.999955	TSPYL2	TSPY-like 2	Multiple_Complex
TC1200010	8.69	7.12	2.96	0.014628	0.991243			NonCoding
TC1200010	5.98	4.41	2.96	0.009198	0.928389	RND1	Rho family GTPase 1	Multiple_Complex
TC2000009	5.38	3.84	2.93	0.003355	0.744794	ZFAS1	ZNFX1 antisense RNA 1	NonCoding
TC0100007	9.82	8.27	2.92	0.00029	0.279023	RNU11	RNA, U11 small nuclear	Multiple_Complex
TC0100013	18.32	16.78	2.91	0.000032	0.097189	RNVU1-18	RNA, variant U1 small nuclear 18; RNA, U1 small nuclear 3; RNA, U1 small nuclear 4; RNA, U1 small nuclear 2; RNA, U1 small nuclear 1	Small_RNA
TC0100013	18.32	16.78	2.91	0.000032	0.097189	RNU1-3	RNA, U1 small nuclear 3	Small_RNA
TC0100015	9.4	7.86	2.91	0.036795	0.999955	LRIF1	ligand dependent nuclear receptor interacting factor 1	Multiple_Complex
TC0100015	18.32	16.78	2.91	0.000032	0.097189	RNVU1-18	RNA, variant U1 small nuclear 18	Small_RNA
TC0600007	14.5	12.96	2.91	0.003722	0.763778	BRD2	bromodomain containing 2	Multiple_Complex
TC1_KI2707	18.32	16.78	2.91	0.000032	0.097189	RNVU1-18	Homo sapiens RNA, variant U1 small nuclear 18 (RNVU1-18), small nuclear RNA.; Homo sapiens RNA, U1 small nuclear 28, pseudogene (RNU1-28P), small nuclea	Small_RNA
TC1400008	18.32	16.78	2.91	0.000032	0.097189	RNU1-28P	RNA, U1 small nuclear 28, pseudogene	Small_RNA
TC0300013	9.45	7.92	2.88	0.001311	0.56359	THUMPD3-AS1	THUMPD3 antisense RNA 1	Multiple_Complex
TC1700010	16.06	14.53	2.88	0.001709	0.605999	SNORD3C	small nucleolar RNA, C/D box 3C	Multiple_Complex
TC0100010	5.99	4.47	2.87	0.007756	0.902202			NonCoding
TC0200012	9.09	7.57	2.87	0.044649	0.999955	seegabo	Transcript Identified by AceView	Unassigned
TC1200010	6.01	4.49	2.87	0.00003	0.097189	RP11-1100L3.8	novel transcript	NonCoding
TC0500011	13.92	12.41	2.86	0.012838	0.963788	LOC647859	occludin pseudogene	Multiple_Complex

TC0600012	17.67	16.17	2.85	0.020828	0.999955			NonCoding
TC0600013	10.89	9.38	2.85	0.008614	0.919926	HIVEP2	human immunodeficiency virus type I enhancer binding protein 2	Multiple_Complex
TC1900008	6.59	5.08	2.85	0.001201	0.543458	kluwy	Transcript Identified by AceView	Unassigned
TC0300008	5.52	4.01	2.84	0.012857	0.963788			NonCoding
TC0600006	10.42	8.91	2.84	0.001091	0.520174	RIPK1	receptor (TNFRSF)-interacting serine-threonine kinase 1	Multiple_Complex
TC0700007	7.31	5.81	2.83	0.000456	0.340164	RNU1-14P	RNA, U1 small nuclear 14, pseudogene [Source:HGNC Symbol;Acc:HGNC:10139]	Multiple_Complex
TC0800009	7.38	5.88	2.83	0.000307	0.289147	HR	hair growth associated	Multiple_Complex
TC0X00009	11.2	9.7	2.83	0.032133	0.999955	PIGA	phosphatidylinositol glycan anchor biosynthesis class A	Multiple_Complex
TC0900007	4.99	3.5	2.82	0.00434	0.781669	RP11-367F23.2	novel transcript; Transcript Identified by AceView	NonCoding
TC0300012	10.68	9.19	2.81	0.000262	0.272297	RNU1-30P	RNA, U1 small nuclear 30, pseudogene [Source:HGNC Symbol;Acc:HGNC:48372]	Small_RNA
TC0900006	5.85	4.36	2.81	0.003884	0.770736			NonCoding
TC1000008	15.38	13.89	2.81	0.023169	0.999955	DUSP5	dual specificity phosphatase 5	Multiple_Complex
TC0100007	12.96	11.48	2.8	0.017947	0.999955	GRHL3	grainyhead-like transcription factor 3	Multiple_Complex
TC0700013	5.12	3.64	2.78	0.000057	0.127325	seybloyby	Transcript Identified by AceView	NonCoding
TC0800008	5.4	3.94	2.76	0.028264	0.999955	TCONS_00014779	Memczak2013 ALT_ACCEPTOR, ncRNA, upstream_start best transcript TCONS_00014779	NonCoding
TC1700009	15.89	14.43	2.76	0.000217	0.247197	SNORD3B-2	small nucleolar RNA, C/D box 3B-2	Multiple_Complex
TC1800007	5.47	4	2.76	0.002247	0.656116	merser	Transcript Identified by AceView	Coding
TC1200007	6.69	5.23	2.75	0.032857	0.999955	RP11-467L13.7	novel transcript, antisense to AMN1	NonCoding
TC0300009	15.21	13.76	2.74	0.002383	0.669795	B3GNT5	UDP-GlcNAc:betaGal beta-1,3-N-acetylglucosaminyltransferase 5	Multiple_Complex
TC0700010	5.79	4.34	2.73	0.01119	0.959571	LOC541472	uncharacterized LOC541472; Transcript Identified by AceView, Entrez Gene ID(s) 256227; 541472; putative novel transcript	NonCoding
TC0800009	5.16	3.71	2.73	0.00415	0.776625	sparzybu	Transcript Identified by AceView	Unassigned
TC0100006	12.72	11.27	2.72	0.002759	0.701493	RNU5E-1	RNA, U5E small nuclear 1	Small_RNA
TC0100014	8.08	6.64	2.72	0.020454	0.999955	garnorbu	Transcript Identified by AceView	Unassigned
TC0300011	4.93	3.48	2.72	0.042464	0.999955	swerslyby	Transcript Identified by AceView	Coding
TC1900010	6.51	5.07	2.72	0.03925	0.999955	KIAA1683	KIAA1683	Multiple_Complex
TC0700006	4.89	3.45	2.71	0.01049	0.950398			NonCoding
TC0200013	5.69	4.26	2.69	0.039316	0.999955			NonCoding
TC0600006	14.62	13.19	2.69	0.000039	0.104367	RNU1-11P	RNA, U1 small nuclear 11, pseudogene [Source:HGNC Symbol;Acc:HGNC:10137]	Small_RNA
TC1200012	7.45	6.02	2.69	0.036883	0.999955	HCAR2	hydroxycarboxylic acid receptor 2	Coding
TC0700012	8.76	7.34	2.68	0.001198	0.543458	RNU1-29P	RNA, U1 small nuclear 29, pseudogene [Source:HGNC Symbol;Acc:HGNC:48371]	Multiple_Complex
TC1100009	8.45	7.03	2.68	0.000046	0.112846	OAF	out at first homolog	NonCoding
TC0100014	4.86	3.45	2.67	0.037425	0.999955	slormubo	Transcript Identified by AceView	Coding
TC0700012	5.28	3.86	2.67	0.000037	0.103191	LHFPL3-AS2	LHFPL3 antisense RNA 2	NonCoding
TC0800008	4.73	3.31	2.67	0.029906	0.999955	steewaby	Transcript Identified by AceView	Coding
TC2200009	4.74	3.33	2.67	0.020944	0.999955			NonCoding
TC0300010	7.76	6.35	2.66	0.001044	0.511807	boydawbo	Transcript Identified by AceView	Unassigned
TC1100009	10.59	9.18	2.66	0.022899	0.999955	OAF	out at first homolog	Coding
TC1200011	6.5	5.09	2.66	0.000832	0.463556	SELPLG	selectin P ligand	Coding
TC0400008	12.52	11.11	2.65	0.005026	0.809372	SGMS2	sphingomyelin synthase 2	Multiple_Complex
TC0400008	8.07	6.66	2.65	0.000156	0.216561	RNU1-138P	RNA, U1 small nuclear 138, pseudogene [Source:HGNC Symbol;Acc:HGNC:48480]	Small_RNA
TC1600007	11.82	10.42	2.65	0.001487	0.571517	ZNF267	zinc finger protein 267	Multiple_Complex
TC0400012	11.55	10.15	2.64	0.021406	0.999955	NIPAL1	NIPA-like domain containing 1	Multiple_Complex
TC0100016	6.12	4.73	2.63	0.045661	0.999955	RP11-12M5.4	novel transcript	NonCoding
TC0100011	12.3	10.91	2.62	0.001821	0.608799	DIEXF	digestive organ expansion factor homolog (zebrafish)	Multiple_Complex
TC0600012	10.96	9.57	2.62	0.002544	0.682184	AKIRIN2	akirin 2	Multiple_Complex
TC0900010	7.62	6.23	2.62	0.019253	0.999955	NINJ1	ninjurin 1	Multiple_Complex
TC0100006	8.36	6.98	2.61	0.0261	0.999955	SLC25A33	solute carrier family 25 (pyrimidine nucleotide carrier), member 33	Multiple_Complex
TC0100018	4.92	3.53	2.61	0.00011	0.173118			NonCoding
TC1000011	4.64	3.25	2.61	0.043535	0.999955			NonCoding
TC1700007	16.75	15.37	2.61	0.000616	0.404263	SNORD3B-1	small nucleolar RNA, C/D box 3B-1; small nucleolar RNA, C/D box 3B-2	Multiple_Complex
TC0200014	7.93	6.56	2.6	0.000421	0.332065	DLX2	distal-less homeobox 2	Coding
TC0300008	7.57	6.19	2.6	0.001801	0.608081	rakuyu	Transcript Identified by AceView	Unassigned
TC0300013	5.23	3.85	2.6	0.000201	0.240745	narmybu	Transcript Identified by AceView	Coding
TC1600007	14.42	13.05	2.6	0.011641	0.959998	HERPUD1	homocysteine-inducible, endoplasmic reticulum stress-inducible, ubiquitin-like domain member 1	Multiple_Complex
TC0100006	5.55	4.18	2.59	0.022148	0.999955			NonCoding
TC0800011	5.7	4.33	2.59	0.033506	0.999955	plywawbu	Transcript Identified by AceView	Coding
TC1800009	7.9	6.53	2.59	0.040468	0.999955			NonCoding
TC0100013	13.41	12.04	2.58	0.006097	0.863736	RNF19B	ring finger protein 19B	Multiple_Complex
TC0100014	4.92	3.55	2.58	0.005936	0.853657			NonCoding
TC0100015	5.34	3.98	2.58	0.004497	0.785232			NonCoding
TC0200015	7.72	6.36	2.58	0.019956	0.999955	steegeybo	Transcript Identified by AceView	Unassigned
TC0300013	6.82	5.46	2.56	0.016314	0.999955	RNU6-1101P	RNA, U6 small nuclear 1101, pseudogene [Source:HGNC Symbol;Acc:HGNC:48064]	Small_RNA
TC0200016	5.58	4.23	2.55	0.007285	0.893879	SPDYA	speedy/RINGO cell cycle regulator family member A	Multiple_Complex
TC1600006	7	5.65	2.55	0.029802	0.999955	klorspar	Transcript Identified by AceView	Unassigned
TC0600007	5.67	4.32	2.54	0.04446	0.999955	MIR548A1	microRNA 548a-1	Precursor_microRNA
TC0900010	14.8	13.46	2.54	0.012961	0.963788	ZFAND5	zinc finger, AN1-type domain 5	Multiple_Complex
TC1000006	5.89	4.54	2.54	0.000509	0.365466	IDI2-AS1	IDI2 antisense RNA 1	NonCoding
TC1200010	8.04	6.69	2.54	0.042168	0.999955	DDIT3	DNA-damage-inducible transcript 3	Coding
TC0900010	12.3	10.96	2.53	0.027787	0.999955	TMEM2	transmembrane protein 2	Multiple_Complex
TC0100009	5.61	4.28	2.52	0.02946	0.999955	fysybo	Transcript Identified by AceView	Coding
TC1000010	6.32	4.99	2.52	0.039413	0.999955	EGR2	early growth response 2	Multiple_Complex
TC1000011	6.01	4.67	2.52	0.001765	0.608081			NonCoding
TC0100009	13.26	11.93	2.51	0.002233	0.65526	RNVU1-15	RNA, variant U1 small nuclear 15	Small_RNA
TC0200008	10.41	9.08	2.51	0.000865	0.463556	BCL2L11	BCL2-like 11 (apoptosis facilitator)	Multiple_Complex
TC0500009	5.77	4.44	2.51	0.005397	0.836345	SQSTM1	Transcript Identified by AceView, Entrez Gene ID(s) 8878	Unassigned
TC1000009	4.62	3.29	2.51	0.04389	0.999955	shopyby	Transcript Identified by AceView	Unassigned
TC1900008	5.15	3.82	2.51	0.027501	0.999955			NonCoding

TC0100011	6.58	5.26	2.5	0.000085	0.156717			NonCoding
TC0200015	4.03	2.71	2.5	0.005527	0.836397	momemo	Transcript Identified by AceView	Unassigned
TC0100017	5.44	4.13	2.48	0.003865	0.770736	RP11-385F5.5	novel transcript; Transcript Identified by AceView	Multiple_Complex
TC0200012	6.78	5.47	2.48	0.019641	0.999955			NonCoding
TC0400008	5.82	4.51	2.48	0.017487	0.999955			NonCoding
TC0700013	6.47	5.16	2.48	0.003587	0.75848	LSMEM1	leucine-rich single-pass membrane protein 1	Multiple_Complex
TC1700009	7.02	5.71	2.48	0.03698	0.999955	MIR22HG	MIR22 host gene	Multiple_Complex
TC1900010	14.7	13.39	2.48	0.007738	0.90202	CTB-50E14.5		Multiple_Complex
TC0200016	16.33	15.02	2.47	0.00749	0.895882	IL1RN	interleukin 1 receptor antagonist	Multiple_Complex
TC1000009	16.45	15.14	2.47	0.002198	0.651407	IDI1	isopentenyl-diphosphate delta isomerase 1	Multiple_Complex
TC1400009	7.67	6.36	2.47	0.000285	0.278446	GCH1	GTP cyclohydrolase 1	Multiple_Complex
TC1600009	5.16	3.86	2.47	0.021709	0.999955			NonCoding
TC1700007	10.67	9.36	2.47	0.03252	0.999955	ARL4D	ADP-ribosylation factor like GTPase 4D	Coding
TC0100011	10.3	9	2.46	0.007462	0.895882	leykarbu	Transcript Identified by AceView	Coding
TC0800008	12.78	11.48	2.46	0.000113	0.174368	TRIB1	tribbles pseudokinase 1	Multiple_Complex
TC0100011	10.49	9.19	2.45	0.015902	0.999955	DYRK3	dual specificity tyrosine-(Y)-phosphorylation regulated kinase 3	Multiple_Complex
TC1100009	7.57	6.28	2.45	0.041664	0.999955	BARX2	BARX homeobox 2	Multiple_Complex
TC1700006	5.64	4.36	2.44	0.010834	0.956227	TMEM88	transmembrane protein 88	Coding
TC0200007	5.47	4.19	2.43	0.003065	0.734859			NonCoding
TC1100011	6.11	4.83	2.43	0.00188	0.616421	zorgleebu	Transcript Identified by AceView	Coding
TC1900007	11.02	9.74	2.43	0.049457	0.999955	IER2	immediate early response 2	Coding
TC0200013	5.91	4.64	2.41	0.008967	0.924384	RNU4-8P	RNA, U4 small nuclear 8, pseudogene [Source:HGNC Symbol;Acc:HGNC:10195]	Small_RNA
TC0300007	9.03	7.76	2.41	0.028142	0.999955	koyfawbo	Transcript Identified by AceView	Unassigned
TC1400009	4.49	3.23	2.41	0.000063	0.134647			NonCoding
TC2100007	4.99	3.72	2.41	0.010578	0.950398	munara	Transcript Identified by AceView	Coding
TC0200016	8.61	7.35	2.4	0.022708	0.999955	SNORA75	small nucleolar RNA, H/ACA box 75	Small_RNA
TC0300011	10.44	9.18	2.4	0.000876	0.466112	VGLL3	vestigial-like family member 3	Multiple_Complex
TC0900006	5.76	4.5	2.4	0.003381	0.744794			NonCoding
TC0X00010	8.5	7.23	2.4	0.000009	0.069027	DNAJA1P3	DnaJ heat shock protein family (Hsp40) member A1 pseudogene 3 [Source:HGNC Symbol;Acc:HGNC:39339]	Pseudogene
TC1100011	4.49	3.23	2.4	0.000023	0.097189	merari	Transcript Identified by AceView	Unassigned
TC0400008	10.5	9.24	2.39	0.000263	0.272297	NOCT	nocturnin	Multiple_Complex
TC0800006	4.89	3.63	2.39	0.036326	0.999955	dargeyby	Transcript Identified by AceView	Coding
TC1700007	16.78	15.52	2.39	0.000626	0.404488	SNORD3A	small nucleolar RNA, C/D box 3A	Small_RNA
TC0200016	12.58	11.33	2.38	0.004957	0.805577	REL	v-rel avian reticuloendotheliosis viral oncogene homolog	Multiple_Complex
TC0500007	5.35	4.11	2.38	0.035492	0.999955	LOC728575	Transcript Identified by AceView, Entrez Gene ID(s) 728575	Coding
TC0700008	7.06	5.81	2.38	0.024763	0.999955			NonCoding
TC0900006	8.33	7.08	2.38	0.006513	0.870775	RP11-363E7.4	novel transcript, overlapping ACER2	NonCoding
TC1100008	4.24	2.99	2.38	0.025393	0.999955	AP002364.1		Precursor_microRNA
TC0300007	9.36	8.12	2.37	0.012806	0.963788	keefawbo	Transcript Identified by AceView	Unassigned
TC0300009	4.3	3.05	2.37	0.019463	0.999955			NonCoding
TC0900007	5.21	3.96	2.37	0.0007	0.418675	flawsher	Transcript Identified by AceView	NonCoding
TC0X00007	6.28	5.04	2.37	0.00806	0.907048	ZNF674-AS1	ZNF674 antisense RNA 1 (head to head)	NonCoding
TC1500007	5.19	3.95	2.37	0.023224	0.999955	wojerby	Transcript Identified by AceView	Unassigned
TC2000008	8.7	7.46	2.37	0.000341	0.297714	RNU1-134P	RNA, U1 small nuclear 134, pseudogene [Source:HGNC Symbol;Acc:HGNC:48476]	Small_RNA
TC0200016	10.9	9.66	2.36	0.008308	0.913881	MXD1	MAX dimerization protein 1	Multiple_Complex
TC0500010	7.24	6	2.36	0.004221	0.779548	RNU6-480P	RNA, U6 small nuclear 480, pseudogene [Source:HGNC Symbol;Acc:HGNC:47443]	Small_RNA
TC0700009	8.91	7.67	2.36	0.000076	0.150802	RNU1-82P	RNA, U1 small nuclear 82, pseudogene [Source:HGNC Symbol;Acc:HGNC:48424]	Small_RNA
TC1400007	8.19	6.95	2.36	0.002092	0.64624	HSPA2	heat shock 70kDa protein 2	Multiple_Complex
TC0100014	5.1	3.87	2.35	0.015702	0.999955	smorplo	Transcript Identified by AceView	Unassigned
TC0900006	9.01	7.78	2.35	0.008321	0.913881	ACER2	alkaline ceramidase 2	Multiple_Complex
TC0100016	9.79	8.56	2.34	0.041543	0.999955	SLC19A2	solute carrier family 19 (thiamine transporter), member 2	Multiple_Complex
TC1700011	5.37	4.14	2.34	0.005431	0.836397	CDC42EP4	CDC42 effector protein (Rho GTPase binding) 4	Multiple_Complex
TC2100006	7.07	5.84	2.34	0.033044	0.999955	kuhora	Transcript Identified by AceView	Coding
TC0100018	10.82	9.6	2.33	0.029681	0.999955	DNAJB4	DnaJ (Hsp40) homolog, subfamily B, member 4	Multiple_Complex
TC0800011	4.21	2.99	2.33	0.018518	0.999955			NonCoding
TC0900010	6.02	4.8	2.33	0.007922	0.902202			NonCoding
TC1100008	5.57	4.35	2.33	0.005785	0.848027	RP11-21L23.2	novel transcript, overlapping to TSKU	NonCoding
TC1600009	7.37	6.15	2.33	0.000445	0.335242	SNHG19	small nucleolar RNA host gene 19	NonCoding
TC0200013	9.7	8.49	2.32	0.03716	0.999955	RNU2-63P	RNA, U2 small nuclear 63, pseudogene [Source:HGNC Symbol;Acc:HGNC:48556]	Small_RNA
TC1200012	7.57	6.36	2.32	0.037975	0.999955	HCAR3	hydroxycarboxylic acid receptor 3	Coding
TC1400010	4.39	3.17	2.32	0.026759	0.999955	florpaw	Transcript Identified by AceView	Coding
TC1700007	6.9	5.69	2.32	0.00384	0.770736	sneewubu	Transcript Identified by AceView	Unassigned
TC0100015	8.87	7.66	2.31	0.000967	0.485943	U1	U1 spliceosomal RNA [Source:RFAM;Acc:RF00003]	Small_RNA
TC0400008	4.95	3.74	2.31	0.004109	0.776625			NonCoding
TC0500009	9.24	8.03	2.31	0.000854	0.463556	ADRB2	adrenoceptor beta 2, surface	Coding
TC1300007	5.25	4.04	2.31	0.004635	0.785827			NonCoding
TC1600009	7.95	6.74	2.31	0.037445	0.999955	slyrar	Transcript Identified by AceView	Unassigned
TC2200009	5.4	4.19	2.31	0.007882	0.902202	APOBEC3A	apolipoprotein B mRNA editing enzyme, catalytic polypeptide-like 3A	Multiple_Complex
TC0100014	4.62	3.42	2.3	0.006569	0.870861			NonCoding
TC1100007	6.66	5.46	2.3	0.002841	0.711573	fawferby	Transcript Identified by AceView	Coding
TC1100012	5.83	4.63	2.3	0.000377	0.310273			NonCoding
TC1200007	6.34	5.14	2.3	0.000247	0.267205	RNU1-69P	RNA, U1 small nuclear 69, pseudogene [Source:HGNC Symbol;Acc:HGNC:48411]	Small_RNA
TC0300008	8.56	7.36	2.29	0.004572	0.785232	C3orf52	chromosome 3 open reading frame 52	Multiple_Complex
TC0100008	4.87	3.69	2.28	0.001785	0.608081	korpla	Transcript Identified by AceView	Coding
TC0100009	5.42	4.23	2.28	0.034955	0.999955	FAM46C	family with sequence similarity 46, member C	Coding
TC0100009	4.32	3.14	2.27	0.001431	0.571517	RP11-763B22.3		Multiple_Complex
TC0100015	3.87	2.68	2.27	0.003153	0.741179			NonCoding

TC0300008	5	3.81	2.27	0.013087	0.963788	RN7SL698P	RNA, 7SL, cytoplasmic 698, pseudogene [Source:HGNC Symbol;Acc:HGNC:46714]	NonCoding
TC0800009	5.5	4.32	2.27	0.029192	0.999955	sazarbu	Transcript Identified by AceView	Coding
TC1300008	16.2	15.02	2.27	0.044355	0.999955	RNU6-70P	RNA, U6 small nuclear 70, pseudogene [Source:HGNC Symbol;Acc:HGNC:42560]	Multiple_Complex
TC0600011	6.32	5.14	2.26	0.006259	0.868734	TMEM217	transmembrane protein 217	Multiple_Complex
TC0800011	8.12	6.94	2.26	0.000402	0.324519	RNU1-35P	RNA, U1 small nuclear 35, pseudogene [Source:HGNC Symbol;Acc:HGNC:48377]	Small_RNA
TC0100015	8.48	7.31	2.25	0.018513	0.999955	S100A3	S100 calcium binding protein A3	Multiple_Complex
TC0200009	6.81	5.64	2.25	0.012002	0.959998	vynerbo	Transcript Identified by AceView	Coding
TC0X00010	11.67	10.5	2.25	0.001553	0.583987	ELF4	E74-like factor 4 (ets domain transcription factor)	Multiple_Complex
TC0500009	8.7	7.54	2.24	0.00254	0.682184	CREBRF	CREB3 regulatory factor	Multiple_Complex
TC1000010	5.83	4.67	2.24	0.017944	0.999955	spawkybu	Transcript Identified by AceView	Coding
TC1400008	8.08	6.92	2.24	0.000785	0.455507	LINC00641	long intergenic non-protein coding RNA 641	NonCoding
TC1800007	4.38	3.22	2.24	0.00989	0.943861	maser	Transcript Identified by AceView	Unassigned
TC0100007	4.85	3.69	2.23	0.028274	0.999955	choyrawbu	Transcript Identified by AceView	NonCoding
TC0100014	4.62	3.47	2.23	0.014211	0.983937			NonCoding
TC0100015	11.1	9.94	2.23	0.013613	0.980319	MIR5087	microRNA 5087	Multiple_Complex
TC0300007	7.23	6.08	2.23	0.04595	0.999955	RP11-80H18.4	novel transcript	NonCoding
TC0300009	4.47	3.32	2.23	0.001371	0.567409	logarbo	Transcript Identified by AceView	Coding
TC1800007	4.92	3.77	2.23	0.046351	0.999955	RNU6-1050P	RNA, U6 small nuclear 1050, pseudogene [Source:HGNC Symbol;Acc:HGNC:48013]	Small_RNA
TC0200010	4.96	3.81	2.22	0.023659	0.999955			NonCoding
TC0300007	3.94	2.79	2.22	0.000274	0.274974			NonCoding
TC1700011	6.35	5.2	2.22	0.032524	0.999955	RNU1-108P	RNA, U1 small nuclear 108, pseudogene [Source:HGNC Symbol;Acc:HGNC:48450]	Small_RNA
TC0100007	12.05	10.91	2.21	0.001389	0.569494	ZDHHC18	zinc finger, DHHC-type containing 18	Multiple_Complex
TC0100009	5.21	4.06	2.21	0.016409	0.999955	AC239800.1		Precursor_microRNA
TC0100009	5.21	4.06	2.21	0.016409	0.999955	AC253578.1		Precursor_microRNA
TC0500008	5.79	4.65	2.21	0.000928	0.481797	CSF2	colony stimulating factor 2 (granulocyte-macrophage)	Coding
TC1400008	6	4.86	2.21	0.0023	0.665687	RNU6-366P	RNA, U6 small nuclear 366, pseudogene [Source:HGNC Symbol;Acc:HGNC:47329]	Small_RNA
TC1600011	6.58	5.44	2.21	0.02823	0.999955	smawsmer	Transcript Identified by AceView	Coding
TC0100013	8.65	7.52	2.2	0.000092	0.165275	STK40	serine/threonine kinase 40	Multiple_Complex
TC0800008	8.66	7.52	2.2	0.000035	0.100368	RNU1-106P	RNA, U1 small nuclear 106, pseudogene [Source:HGNC Symbol;Acc:HGNC:48448]	Small_RNA
TC1100007	4.59	3.45	2.2	0.00258	0.688164	RNU6-99P	RNA, U6 small nuclear 99, pseudogene [Source:HGNC Symbol;Acc:HGNC:47062]	Small_RNA
TC1300007	4.84	3.7	2.2	0.008511	0.916101			NonCoding
TC1400006	7.5	6.36	2.2	0.041163	0.999955	TRAJ8	T cell receptor alpha joining 8	Pseudogene
TC0100016	13.23	12.09	2.19	0.003276	0.744794	ABL2	ABL proto-oncogene 2, non-receptor tyrosine kinase	Multiple_Complex
TC0500007	7.82	6.69	2.19	0.01654	0.999955	shazerby	Transcript Identified by AceView	Coding
TC0700009	5.28	4.15	2.19	0.02961	0.999955			NonCoding
TC1100011	4.45	3.32	2.19	0.027568	0.999955			NonCoding
TC2000007	10.25	9.12	2.19	0.016525	0.999955	RNU6-1251P	RNA, U6 small nuclear 1251, pseudogene [Source:HGNC Symbol;Acc:HGNC:48214]	Small_RNA
TC0100016	5.22	4.09	2.18	0.01848	0.999955			NonCoding
TC0300013	4.7	3.57	2.18	0.019234	0.999955	glokeebu	Transcript Identified by AceView	NonCoding
TC0900009	6.62	5.5	2.18	0.0347	0.999955	RP11-513M16.7	novel transcript, antisense to DENND4C	NonCoding
TC1200007	4.66	3.54	2.18	0.008327	0.913881			NonCoding
TC1400007	6.51	5.38	2.18	0.04357	0.999955	shopar	Transcript Identified by AceView	Unassigned
TC0100009	15.09	13.97	2.17	0.000275	0.274974	RNVU1-14	RNA, variant U1 small nuclear 14	Multiple_Complex
TC0100015	6.12	5.01	2.17	0.01454	0.989137	yukayo	Transcript Identified by AceView	Unassigned
TC0100016	5.46	4.34	2.17	0.043944	0.999955	swymubu	Transcript Identified by AceView	Coding
TC0500007	8.3	7.18	2.17	0.04348	0.999955	floystobu	Transcript Identified by AceView	Unassigned
TC1200007	6.46	5.35	2.17	0.031274	0.999955	nuner	Transcript Identified by AceView	Unassigned
TC1600009	10.09	8.97	2.17	0.004036	0.775973	gersnu	Transcript Identified by AceView	Unassigned
TC1600011	6.93	5.81	2.17	0.000122	0.184119	RNU1-103P	RNA, U1 small nuclear 103, pseudogene [Source:HGNC Symbol;Acc:HGNC:48445]	Small_RNA
TC0100015	6.94	5.82	2.16	0.044618	0.999955	RNVU1-8	RNA, variant U1 small nuclear 8	Multiple_Complex
TC0500012	5.68	4.57	2.16	0.039564	0.999955			NonCoding
TC1000009	14.96	13.85	2.16	0.000005	0.068513	BAG3	BCL2-associated athanogene 3	Coding
TC1300008	5.23	4.11	2.16	0.035294	0.999955			NonCoding
TC1900008	8.57	7.46	2.16	0.001726	0.606851	MED29	mediator complex subunit 29	Multiple_Complex
TC2200007	5.3	4.19	2.16	0.00031	0.290081	HMOX1	heme oxygenase 1	Multiple_Complex
TC0400008	6.07	4.96	2.15	0.000939	0.481797	RNU6-531P	RNA, U6 small nuclear 531, pseudogene [Source:HGNC Symbol;Acc:HGNC:47494]	Small_RNA
TC0500009	5.8	4.7	2.15	0.006372	0.868734			NonCoding
TC1100011	7.36	6.25	2.15	0.00414	0.776625	RP11-770G2.5	novel transcript	NonCoding
TC1600010	4.92	3.82	2.15	0.011797	0.959998			NonCoding
TC1800007	5.66	4.56	2.15	0.027448	0.999955	volo	Transcript Identified by AceView	Coding
TC1800007	8.12	7.01	2.15	0.023705	0.999955	sporry	Transcript Identified by AceView	NonCoding
TC0100016	13.82	12.72	2.14	0.000903	0.47326	ETV3	ets variant 3	Multiple_Complex
TC0100017	4.86	3.76	2.14	0.018102	0.999955	korlawbo	Transcript Identified by AceView	Coding
TC0100018	8.4	7.31	2.14	0.000265	0.272811	HNRNPU-AS1	Homo sapiens HNRNPU antisense RNA 1 (HNRNPU-AS1), long non-coding RNA.; HNRNPU antisense RNA 1 [Source:HGNC Symbol;Acc:HGNC:27651]; HNRNPU ant	NonCoding
TC0200007	5.89	4.8	2.14	0.017925	0.999955			NonCoding
TC0400012	7.41	6.31	2.14	0.035054	0.999955	chargar	Transcript Identified by AceView; putative novel transcript	NonCoding
TC0700008	4.61	3.52	2.14	0.003379	0.744794			NonCoding
TC1100007	8	6.9	2.14	0.035377	0.999955	RP1-68D18.2	novel transcript	NonCoding
TC2000007	4.82	3.72	2.14	0.048488	0.999955	gorzaw	Transcript Identified by AceView	Coding
TSUnmapp	4.23	3.13	2.14	0.012699	0.963788			NonCoding
TC0100010	6.87	5.78	2.13	0.008327	0.913881	jeyreebu	Transcript Identified by AceView	Coding
TC0300011	4.46	3.37	2.13	0.010939	0.957813			NonCoding
TC0800008	4.95	3.86	2.13	0.024319	0.999955			NonCoding
TC1100009	3.67	2.59	2.13	0.03808	0.999955	teyglubu	Transcript Identified by AceView	Coding
TC1500007	4.05	2.96	2.13	0.047879	0.999955	hosoru	Transcript Identified by AceView	Coding
TC1700007	8.88	7.79	2.13	0.005698	0.84631	MAP2K3	mitogen-activated protein kinase kinase 3	Multiple_Complex
TC0100009	8.18	7.1	2.12	0.021537	0.999955	RP11-196G18.24	novel transcript	NonCoding

TC0100010	8.5	7.42	2.12	0.010517	0.950398	EIF1P3	eukaryotic translation initiation factor 1 pseudogene 3 [Source:HGNC Symbol;Acc:HGNC:49616]	Pseudogene
TC0200011	7.22	6.14	2.12	0.010107	0.950301			NonCoding
TC0500009	5.67	4.58	2.12	0.015179	0.995547	MIR146A	microRNA 146a	Multiple_Complex
TC0800009	6.91	5.82	2.12	0.000427	0.334608	RNU1-148P	RNA, U1 small nuclear 148, pseudogene [Source:HGNC Symbol;Acc:HGNC:48490]	Small_RNA
TC1000008	11.5	10.42	2.12	0.00357	0.757209	NFKB2	nuclear factor of kappa light polypeptide gene enhancer in B-cells 2 (p49/p100)	Multiple_Complex
TC1000011	5.57	4.49	2.12	0.013367	0.975019			NonCoding
TC1300007	6.45	5.36	2.12	0.035979	0.999955	LINC00370	long intergenic non-protein coding RNA 370 [Source:HGNC Symbol;Acc:HGNC:42692]; putative novel transcript	NonCoding
TC2000007	5.44	4.36	2.12	0.011269	0.959571	RNU4-40P	RNA, U4 small nuclear 40, pseudogene [Source:HGNC Symbol;Acc:HGNC:46976]	Small_RNA
TC0100009	5.63	4.55	2.11	0.009693	0.938059	spuply	Transcript Identified by AceView	Coding
TC0300008	4.81	3.73	2.11	0.035828	0.999955	tiwamo	Transcript Identified by AceView	Coding
TC0Y00007	6.62	5.54	2.11	0.032659	0.999955	TTTY14andNCRNA00185	Transcript Identified by AceView, Entrez Gene ID(s) 55410; 83869	Unassigned
TC1000008	17.26	16.18	2.11	0.010639	0.950398	PLAU	plasminogen activator, urokinase	Multiple_Complex
TC1100009	11.59	10.52	2.11	0.025027	0.999955	PHLDA2	pleckstrin homology-like domain, family A, member 2	Multiple_Complex
TC1500009	11.58	10.51	2.11	0.000518	0.370185	TLE3	transducin-like enhancer of split 3	Multiple_Complex
TC1600007	10.03	8.96	2.11	0.010131	0.950301			NonCoding
TC0100011	4.77	3.7	2.1	0.032864	0.999955			NonCoding
TC0500007	5.83	4.76	2.1	0.017859	0.999955			NonCoding
TC0900012	4.42	3.35	2.1	0.000785	0.455507	RP13-122B23.8	novel transcript, overlapping NRARP	NonCoding
TC0X00007	6.17	5.09	2.1	0.007166	0.889245			NonCoding
TC1700011	10.5	9.43	2.1	0.000052	0.121515	ERN1	endoplasmic reticulum to nucleus signaling 1	Multiple_Complex
TC1900011	5.11	4.04	2.1	0.038819	0.999955	PSG7	pregnancy specific beta-1-glycoprotein 7 (gene/pseudogene)	Multiple_Complex
TC0100013	5.71	4.65	2.09	0.011962	0.959998			NonCoding
TC0100015	12.77	11.71	2.09	0.006359	0.868734	RNVU1-19	RNA, variant U1 small nuclear 19	Small_RNA
TC0300013	7.18	6.11	2.09	0.021019	0.999955	wysworby	Transcript Identified by AceView	Coding
TC0500012	6.29	5.22	2.09	0.011573	0.959998	rohuyu	Transcript Identified by AceView	Coding
TC0600006	5.25	4.19	2.09	0.030993	0.999955			NonCoding
TC0600012	4.76	3.7	2.09	0.007804	0.902202	shofluby	Transcript Identified by AceView	Coding
TC1000010	6.54	5.48	2.09	0.011376	0.959571	leemorby	Transcript Identified by AceView	Unassigned
TC0300008	4.08	3.02	2.08	0.008354	0.913881			NonCoding
TC0500007	4.68	3.62	2.08	0.01998	0.999955	rawplawby	Transcript Identified by AceView	Coding
TC0600012	6.23	5.17	2.08	0.046499	0.999955	bujor	Transcript Identified by AceView	Unassigned
TC0800010	5.77	4.71	2.08	0.013046	0.963788	SNORA1	Small nucleolar RNA SNORA1 [Source:RFAM;Acc:RF00408]	Small_RNA
TC1600006	5.31	4.25	2.08	0.000687	0.416247	RNU1-22P	RNA, U1 small nuclear 22, pseudogene [Source:HGNC Symbol;Acc:HGNC:41963]	Small_RNA
TC1900011	6.85	5.79	2.08	0.000962	0.485943			NonCoding
TSUnmapp	5.31	4.25	2.08	0.000687	0.416247			Small_RNA
TC0500011	10.31	9.26	2.07	0.044642	0.999955	FEM1C	fem-1 homolog c (C. elegans)	Multiple_Complex
TC0600011	5.52	4.46	2.07	0.010945	0.957813	HIST1H1T	histone cluster 1, H1t	Coding
TC1100013	11.92	10.88	2.07	0.003264	0.744794	STX3	syntaxin 3	Multiple_Complex
TC0100013	10.65	9.61	2.06	0.003652	0.763778	RSRP1	arginine/serine-rich protein 1	Multiple_Complex
TC0100018	13.57	12.53	2.06	0.000013	0.077608	SFPQ	splicing factor proline/glutamine-rich	Multiple_Complex
TC0700012	5.93	4.89	2.06	0.026584	0.999955			NonCoding
TC1300007	4.31	3.27	2.06	0.00664	0.873491			NonCoding
TC1300008	15.04	14	2.06	0.002992	0.724087	HSPH1	heat shock 105kDa/110kDa protein 1	Multiple_Complex
TC1600006	6.07	5.02	2.06	0.033193	0.999955	CTD-2270P14.2	tec	Unassigned
TC0200013	16.85	15.81	2.05	0.041643	0.999955	IL1B	interleukin 1 beta	Multiple_Complex
TC0300006	6.11	5.07	2.05	0.000162	0.217793	RNU1-96P	RNA, U1 small nuclear 96, pseudogene [Source:HGNC Symbol;Acc:HGNC:48438]	Small_RNA
TC0300011	4.36	3.32	2.05	0.043244	0.999955	LINC00696	long intergenic non-protein coding RNA 696	NonCoding
TC0700006	4.29	3.25	2.05	0.03289	0.999955	sarshybu	Transcript Identified by AceView	Coding
TC1100008	7.03	5.99	2.05	0.024051	0.999955	zorkarby	Transcript Identified by AceView	Unassigned
TC1100008	3.86	2.82	2.05	0.018401	0.999955			NonCoding
TC1100010	4.54	3.51	2.05	0.029354	0.999955	LOC440028	uncharacterized LOC440028; novel transcript	NonCoding
TC1100011	4.92	3.89	2.05	0.000026	0.097189	RP11-178H8.7	novel transcript	NonCoding
TC1300007	4.64	3.61	2.05	0.029467	0.999955			NonCoding
TC1700009	6.04	5.01	2.05	0.003156	0.741179	RP11-667K14.3	novel transcript	NonCoding
TC1700012	6.3	5.27	2.05	0.000544	0.380744	CDK12	cyclin-dependent kinase 12	NonCoding
TC0100007	4.21	3.19	2.04	0.005875	0.851618	sporawbo	Transcript Identified by AceView	Unassigned
TC0200010	6.74	5.72	2.04	0.029499	0.999955			NonCoding
TC0900006	11.88	10.85	2.04	0.001966	0.627868	LURAP1L	leucine rich adaptor protein 1-like	Multiple_Complex
TC0100009	8.46	7.44	2.03	0.011637	0.959998	U1	U1 spliceosomal RNA [Source:RFAM;Acc:RF00003]	Multiple_Complex
TC0100011	7.53	6.52	2.03	0.00044	0.334608	TRAF3IP3	TRAF3 interacting protein 3	Multiple_Complex
TC0200015	5.1	4.08	2.03	0.021975	0.999955			NonCoding
TC0500010	6.05	5.02	2.03	0.019418	0.999955	waslawbu	Transcript Identified by AceView	Coding
TC0600007	13.46	12.44	2.03	0.015013	0.99496	SRSF3	serine/arginine-rich splicing factor 3	Multiple_Complex
TC0X00007	8.89	7.87	2.03	0.002907	0.717301	sperfor	Transcript Identified by AceView	Coding
TC1000011	5.05	4.03	2.03	0.042714	0.999955			NonCoding
TC1100007	6.41	5.38	2.03	0.030856	0.999955	AP001350.4	TEC	NonCoding
TC1500009	8.6	7.58	2.03	0.011557	0.959998	RNU1-78P	RNA, U1 small nuclear 78, pseudogene [Source:HGNC Symbol;Acc:HGNC:48420]	Small_RNA
TC1600009	6.83	5.81	2.03	0.021237	0.999955	CRYM	crystallin mu	Multiple_Complex
TC1900007	4.95	3.93	2.03	0.003563	0.75685	FARSA-AS1	FARSA antisense RNA 1 [Source:HGNC Symbol;Acc:HGNC:50479]; novel transcript, antisense to FARSA	NonCoding
TC0200010	4.85	3.84	2.02	0.024031	0.999955			NonCoding
TC0400008	9.93	8.92	2.02	0.00284	0.711573	USP38	ubiquitin specific peptidase 38	Multiple_Complex
TC0400010	5.74	4.73	2.02	0.043569	0.999955	RNU1-63P	RNA, U1 small nuclear 63, pseudogene [Source:HGNC Symbol;Acc:HGNC:48405]	Small_RNA
TC0500006	5.43	4.42	2.02	0.007069	0.889245	myskoby	Transcript Identified by AceView	Unassigned
TC0500009	5.03	4.02	2.02	0.044746	0.999955			NonCoding
TC0800008	10.75	9.73	2.02	0.018142	0.999955	gashoy	Transcript Identified by AceView	Unassigned
TC0900010	5.83	4.81	2.02	0.037276	0.999955	RNA5SP290	RNA, 5S ribosomal pseudogene 290 [Source:HGNC Symbol;Acc:HGNC:43190]	Ribosomal
TC0200016	5.87	4.86	2.01	0.012558	0.962949			NonCoding

TC0300012	6.08	5.08	2.01	0.005114	0.815406	roremo	Transcript Identified by AceView	NonCoding
TC0400006	4.5	3.49	2.01	0.000367	0.310175	chufley	Transcript Identified by AceView	Unassigned
TC0600008	6.38	5.37	2.01	0.010136	0.950301	RNU1-136P	RNA, U1 small nuclear 136, pseudogene [Source:HGNC Symbol;Acc:HGNC:48478]	Small_RNA
TC0900007	11.5	10.49	2.01	0.001084	0.520174	TLE4	transducin-like enhancer of split 4	Multiple_Complex
TC1800006	6.62	5.61	2.01	0.016703	0.999955	klawrer	Transcript Identified by AceView	Coding
TC2100006	4.16	3.15	2.01	0.045107	0.999955			NonCoding
TC0X00010	4.43	5.44	-2.01	0.00703	0.889245	MIR503	microRNA 503	Precursor_microRNA
TC1900012	5.27	6.28	-2.01	0.001221	0.548908	ZNF772	zinc finger protein 772	Multiple_Complex
TC0500011	7.5	8.51	-2.02	0.001473	0.571517	GIN1	gypsy retrotransposon integrase 1	Multiple_Complex
TC1300009	3.95	4.96	-2.02	0.011054	0.957813			NonCoding
TC1600006	5.06	6.08	-2.02	0.041005	0.999955	CCNF	cyclin F	Multiple_Complex
TC2200006	3.32	4.33	-2.02	0.000583	0.394441			NonCoding
TC0300012	4.22	5.23	-2.03	0.016368	0.999955	RAB43	Memczak2013 ALT_ACCEPTOR, ALT_DONOR, coding, INTERNAL, intronic best transcript NM_001204883	NonCoding
TC0600013	3.36	4.38	-2.03	0.008474	0.915165			NonCoding
TC1300007	4.37	5.39	-2.03	0.034762	0.999955	TNFSF13B	Transcript Identified by AceView, Entrez Gene ID(s) 10673	Unassigned
TC1500007	3.63	4.65	-2.03	0.014652	0.992035	zeester	Transcript Identified by AceView	Coding
TC1700009	3.01	4.03	-2.03	0.023024	0.999955			NonCoding
TC0100013	4.32	5.35	-2.04	0.021036	0.999955	AGMAT	agmatinase	Coding
TC0400008	8.46	9.49	-2.04	0.003291	0.744794	MMAA	methylmalonic aciduria (cobalamin deficiency) cblA type	Multiple_Complex
TC0700008	3.19	4.22	-2.04	0.025933	0.999955	spawsmoybu	Transcript Identified by AceView	Coding
TC0500010	2.78	3.82	-2.05	0.0011	0.520174	snoystobu	Transcript Identified by AceView	Coding
TC1100007	3.29	4.33	-2.05	0.045989	0.999955	glerplu	Transcript Identified by AceView	Coding
TC0100014	5.64	6.68	-2.06	0.02343	0.999955	chaplo	Transcript Identified by AceView	Coding
TC0200012	8.72	9.77	-2.06	0.015677	0.999955	DHX57	DEAH (Asp-Glu-Ala-Asp/His) box polypeptide 57	Multiple_Complex
TC0900008	5.71	6.76	-2.06	0.001946	0.625996	stysoyby	Transcript Identified by AceView	Unassigned
TC1000011	6.23	7.27	-2.06	0.036346	0.999955	RP11-427L15.2	novel transcript sense intonic to CACUL1	NonCoding
TC1200008	9.85	10.89	-2.06	0.032006	0.999955			NonCoding
TC0700006	4.08	5.13	-2.07	0.011985	0.959998	AC006042.8		Pseudogene
TC1200008	7.38	8.43	-2.07	0.014633	0.991243	flonu	Transcript Identified by AceView	Unassigned
TC0200013	9	10.06	-2.08	0.001841	0.608799	REV1	REV1, DNA directed polymerase	Multiple_Complex
TC1200008	5.44	6.49	-2.08	0.005476	0.836397	AC144522.1		Precursor_microRNA
TC1700010	9.74	10.8	-2.08	0.030002	0.999955	sormu	Transcript Identified by AceView	Unassigned
TC1900007	3.41	4.46	-2.08	0.014506	0.989069	speechee	Transcript Identified by AceView	Coding
TC0100013	5.43	6.49	-2.09	0.010184	0.950301	cholorbu	Transcript Identified by AceView	Coding
TC0200013	5.45	6.51	-2.09	0.028797	0.999955	PLGLB1	Transcript Identified by AceView, Entrez Gene ID(s) 285074; 5343	Unassigned
TC0500010	3.06	4.12	-2.09	0.004759	0.792647	geystubu	Transcript Identified by AceView	Unassigned
TC2000006	6.93	8	-2.09	0.007776	0.902202	PET117	PET117 homolog; CSRP2 binding protein	Multiple_Complex
TC0300012	5.18	6.25	-2.1	0.016732	0.999955	klybeebu	Transcript Identified by AceView	Coding
TC0700010	3.71	4.78	-2.1	0.031475	0.999955	neegloyby	Transcript Identified by AceView	Unassigned
TC1400009	6.49	7.56	-2.1	0.019079	0.999955	blorzer	Transcript Identified by AceView	Unassigned
TC0100013	5.28	6.36	-2.11	0.035899	0.999955	moylybo	Transcript Identified by AceView	Unassigned
TC0200009	6.72	7.8	-2.11	0.01786	0.999955	swarfeebu	Transcript Identified by AceView	Unassigned
TC0400009	4.89	5.97	-2.11	0.045048	0.999955			NonCoding
TC0400011	2.66	3.75	-2.13	0.002834	0.711573	LOC100131611	Transcript Identified by AceView, Entrez Gene ID(s) 100131611	Unassigned
TC0900010	6.08	7.17	-2.13	0.010792	0.954212	ZNF484	zinc finger protein 484	Multiple_Complex
TC2200009	5.87	6.96	-2.13	0.000501	0.361836	CDPF1	cysteine rich, DPF motif domain containing 1	Multiple_Complex
TC1400007	6.45	7.54	-2.14	0.047429	0.999955	tehiri	Transcript Identified by AceView	Coding
TC0100007	5.42	6.52	-2.15	0.020756	0.999955	warrarbo	Transcript Identified by AceView	Unassigned
TC0200013	3.63	4.74	-2.15	0.010569	0.950398			NonCoding
TC0900006	4.61	5.71	-2.15	0.009374	0.930282	RNU7-185P	RNA, U7 small nuclear 185 pseudogene [Source:HGNC Symbol;Acc:HGNC:45719]	Small_RNA
TC1900008	5.99	7.1	-2.15	0.010878	0.957813	ZNF221	zinc finger protein 221	Multiple_Complex
TC1500009	11.57	12.69	-2.16	0.010808	0.954567	RFX7	regulatory factor X, 7	Multiple_Complex
TC0500009	5.6	6.72	-2.17	0.005366	0.834469	ZNF354C	zinc finger protein 354C	Coding
TC0900010	4.58	5.7	-2.17	0.019713	0.999955	RP11-401G5.1		Multiple_Complex
TC1500009	8.45	9.57	-2.17	0.039583	0.999955	TTBK2	tau tubulin kinase 2	Multiple_Complex
TC0700012	4.82	5.94	-2.18	0.002072	0.64624			NonCoding
TC2100007	4.01	5.14	-2.19	0.001255	0.550607			NonCoding
TC0400008	4.35	5.48	-2.2	0.006189	0.866527			NonCoding
TC0X00010	6.04	7.2	-2.23	0.003269	0.744794	AL772400.1		Precursor_microRNA
TC1300008	10.08	11.25	-2.25	0.002444	0.675789	CHAMP1	chromosome alignment maintaining phosphoprotein 1	Multiple_Complex
TC0400009	6.73	7.91	-2.26	0.045855	0.999955	bluspuby	Transcript Identified by AceView	Unassigned
TC0100017	3.25	4.43	-2.27	0.022764	0.999955			NonCoding
TC0800008	8.07	9.25	-2.27	0.047999	0.999955	PLEKHF2	pleckstrin homology domain containing, family F (with FYVE domain) member 2	Multiple_Complex
TC0400011	5.74	6.93	-2.28	0.017379	0.999955	snorkloby	Transcript Identified by AceView	Unassigned
TC1200007	4.8	5.99	-2.28	0.027405	0.999955	RP11-274J7.2	novel transcript	NonCoding
TC1900009	5.65	6.84	-2.29	0.000867	0.463556	waflee	Transcript Identified by AceView	Unassigned
TC0400007	4.56	5.76	-2.3	0.022634	0.999955			NonCoding
TC0300011	5.24	6.45	-2.31	0.031079	0.999955	MAP4	Transcript Identified by AceView, Entrez Gene ID(s) 4134	Unassigned
TC1900011	6.08	7.32	-2.36	0.000997	0.495821	ZNF420	zinc finger protein 420	Multiple_Complex
TC0300011	4.45	5.7	-2.37	0.004092	0.776625	nupoybu	Transcript Identified by AceView	Coding
TC0900008	5.15	6.4	-2.37	0.014302	0.983937	sawseeby	Transcript Identified by AceView	Coding
TC0900011	3.39	4.65	-2.38	0.028832	0.999955	foshor	Transcript Identified by AceView	Unassigned
TC0200014	6.23	7.49	-2.4	0.012009	0.959998	lalobo	Transcript Identified by AceView	Unassigned
TC0200013	5.1	6.39	-2.45	0.002494	0.676213	towaro	Transcript Identified by AceView	Unassigned
TC0400008	4.45	5.75	-2.46	0.011618	0.959998	TIGD2	tigger transposable element derived 2	Multiple_Complex
TC0100007	4.46	5.77	-2.47	0.041041	0.999955			NonCoding
TC0300009	4.51	5.82	-2.47	0.000333	0.295523	RN7SKP296	RNA, 7SK small nuclear pseudogene 296 [Source:HGNC Symbol;Acc:HGNC:46020]	NonCoding

TC1100011	5.31	6.64	-2.51	0.005281	0.832854	chachor	Transcript Identified by AceView	Unassigned
TC0100009	4.04	5.39	-2.54	0.009008	0.924384	chawber	Transcript Identified by AceView	Coding
TC0100010	3.69	5.05	-2.55	0.02896	0.999955	RNU7-183P	RNA, U7 small nuclear 183 pseudogene [Source:HGNC Symbol;Acc:HGNC:45717]	Small_RNA
TC0900007	4.65	6	-2.55	0.033666	0.999955			NonCoding
TC1200007	4.72	6.07	-2.55	0.014999	0.99496	zerblobu	Transcript Identified by AceView	Unassigned
TC0200008	3.58	4.96	-2.6	0.028578	0.999955	hitomo	Transcript Identified by AceView	Coding
TC1700010	6.56	7.93	-2.6	0.015958	0.999955	spoyslu	Transcript Identified by AceView	Coding
TC1500008	9.11	10.51	-2.65	0.004182	0.776625			NonCoding
TC0300014	7.76	9.17	-2.66	0.040326	0.999955	TBCCD1	TBCC domain containing 1	Multiple_Complex
TC1200012	5.13	6.54	-2.66	0.001672	0.598242	dyblerbu	Transcript Identified by AceView	Unassigned
TC0200014	3.74	5.16	-2.67	0.007586	0.896965			NonCoding
TC0400011	3.85	5.29	-2.72	0.003379	0.744794	skarsmoyby	Transcript Identified by AceView	Unassigned
TC0300009	4.88	6.33	-2.73	0.015553	0.999955	snerdarbu	Transcript Identified by AceView	Unassigned
TC0800009	7.03	8.48	-2.73	0.028676	0.999955	ZNF623	zinc finger protein 623	Coding
TC0600013	3.9	5.42	-2.86	0.001076	0.520174	MIR3145	microRNA 3145	Precursor_microRNA
TC1000007	4.36	5.89	-2.88	0.00448	0.785232	goyfor	Transcript Identified by AceView	Unassigned
TSUnmappe	7.51	9.05	-2.91	0.003519	0.753392	CCDC84	coiled-coil domain containing 84	NonCoding
TC0100009	5.58	7.17	-3.01	0.043762	0.999955	MIR553	microRNA 553	Precursor_microRNA
TC1200009	9.19	10.79	-3.03	0.001153	0.530422	DCP1B	decapping mRNA 1B	Multiple_Complex
TC1200011	7.37	9.06	-3.22	0.033551	0.999955			NonCoding
TC0X00008	5.9	7.65	-3.36	0.001784	0.608081	ZNF449	zinc finger protein 449	Coding
TC0100012	7.68	9.45	-3.41	0.01483	0.994737	reeplu	Transcript Identified by AceView	Unassigned
TC0500008	4.57	6.35	-3.42	0.002905	0.717301	jerflee	Transcript Identified by AceView	Unassigned
TC0200014	8.03	9.92	-3.7	0.006458	0.868734	vugloy	Transcript Identified by AceView	Unassigned
TC0100012	7.09	9.05	-3.9	0.00534	0.834256	niroma	Transcript Identified by AceView	Unassigned
TC0600010	6.26	8.23	-3.92	0.002913	0.717301	merblor	Transcript Identified by AceView	Unassigned
TC0200014	8.88	10.86	-3.95	0.001365	0.567409	fuder	Transcript Identified by AceView	Unassigned
TC1000011	5.04	7.21	-4.53	0.010544	0.950398	zusner	Transcript Identified by AceView	Coding
TC0400008	7.44	9.64	-4.61	0.000596	0.396695	SPRY1	sprouty RTK signaling antagonist 1	Multiple_Complex
TC0M0000	6.39	8.71	-4.98	0.028656	0.999955	MT-TD	mitochondrially encoded tRNA aspartic acid [Source:HGNC Symbol;Acc:HGNC:7478]	Multiple_Complex
TC0900011	5.53	8.12	-6.04	0.01106	0.957813			NonCoding
