# Supplementary material for: Notch signalling is a potential resistance mechanism of progenitor cells within patient‐derived prostate cultures following ROS‐inducing treatments
Source: FEBS Lett. 2019 Sep 17;594(2):209–26. doi: 10.1002/1873-3468.13589 (PMC7003772; doi:10.1002/1873-3468.13589)
Supplement: Supplementary file 15 [file FEB2-594-209-s015.docx]

Table S1 - Patient information of all cell cultures used in the study

Table S2 - Antibodies used in the study

Table S3 - All samples 'treated against untreated' Excel file

Table S4 - Taqman probes used in the study

Table S5 - LIMMA meta-analysis of ALL samples 'treated against untreated' Excel file
